# Brain TRPV1 channel-mediated calcium influx: the immunomodulatory pathway of acupuncture in neuroinflammation

**DOI:** 10.3389/fimmu.2025.1700282

**Published:** 2025-12-11

**Authors:** Qingqing Tang, Min He, Peng Zheng, Mengmeng Sun, Jiazhen Cao, Qi Zhang, Jing He, Run Sun, Bing Zhu, Tie Li

**Affiliations:** 1 Department of Acupuncture and Tuina, Changchun University of Chinese Medicine, Changchun, Jilin, China; 2 Northeast Asia Research Institute of Traditional Chinese Medicine, Changchun University of Chinese Medicine, Changchun, Jilin, China; 3 Department of Rehabilitation, The Third Affiliated Hospital of Changchun University of Chinese Medicine, Changchun, China; 4 School of Nursing, Changchun University of Chinese Medicine, Changchun, Jilin, China; 5 College of Acupuncture and Orthopedics, Hubei University of Chinese Medicine, Wuhan, Hubei, China; 6 Institute of Acupuncture and Moxibustion, China Academy of Chinese Medical Sciences, Beijing, China

**Keywords:** brain dysfunction, immunoregulation, microglia, neuroinflammation, TRPV1 channel

## Abstract

Neuroinflammation represents the central pathological process in neurological disorders. Effectively regulating neuroinflammation to restore immune homeostasis and alleviate neuronal damage has emerged as a critical strategy in the prevention and treatment of these diseases. In recent years, the role of acupuncture in neuroimmune regulation, along with its anti-inflammatory and analgesic effects, has attracted considerable attention. Its potential to modulate immune homeostasis and inflammatory responses through various targets and pathways has been gradually elucidated, offering new research directions for the regulation of neuroinflammation. A series of studies have emphasized that acupuncture has significant clinical applications by regulating the immunoinflammatory pathway mediated by the brain’s TRPV1 channel. This discovery not only enhances the scientific understanding of the mechanisms underlying acupuncture but also offers new potential targets for the prevention and treatment of neuroinflammation-related diseases. The immunomodulatory properties of brain TRPV1 channels in inflammation associated with the nervous system have been emphasized. Furthermore, this study explores the immunomodulatory benefits of acupuncture in treating neuroinflammation, focusing on the potential mechanisms of TRPV1 channels at the brain level, as well as the criteria for selecting acupoints, intensity, frequency, and other relevant parameters in these studies. A deeper understanding of the neuroimmune regulatory mechanisms mediated by brain TRPV1 channels may offer new strategies and approaches for developing treatments or preventing neuropathological diseases.

## 1 Introduction

Neuroinflammation is a specific immune response of the central nervous system (CNS) to injury, infection, or pathological stimuli. Moderate inflammatory responses can exert protective effects by eliminating pathogens, phagocytosing cellular debris, and maintaining microenvironmental homeostasis. However, excessive or persistent neuroinflammation can lead to immune hyperactivation in the CNS, further damaging the brain and causing pathological alterations in brain structure and function. It is characterized by the abnormal activation of microglia and astrocytes, a cascade release of pro-inflammatory mediators, and the upregulation of neurotoxic pathways. The resulting chronic inflammatory microenvironment not only disrupts the blood-brain barrier (BBB) and promotes the infiltration of peripheral immune cells but also induces pathological changes such as mitochondrial dysfunction in brain neurons and synaptic plasticity impairment, leading to the malignant progression of diseases (1, 2). It is noteworthy that a significant degree of neuroinflammation is closely associated with damage to both the peripheral nervous system (PNS) and the CNS. Persistent neuroinflammation is a key factor in the pathogenesis of neurological disorders, driving disease progression and causing brain damage (3–5). Meanwhile, central sensitization, as a compensatory response of the nervous system to peripheral and central neural lesions, manifests as an overexcited state of the CNS. The nociceptive transmission signals it mediates can further activate the inflammatory cascade in the CNS, and the resulting neuroinflammatory response can exacerbate the pathological changes associated with central sensitization, ultimately leading to peripheral pain hypersensitivity in the corresponding spinal cord or brain regions, as well as the systemic spread of inflammation (6–10). The pathological interplay between neuroinflammation, neurological damage, and central sensitization, coupled with the crosstalk between peripheral and central immunity, forms a vicious cycle that exacerbates pathological alterations in the nervous system and leads to irreversible damage to the brain. This situation has emerged as a significant challenge in the global public health domain.

According to the 2021 Global Burden of Disease Study, neurological disorders have emerged as the leading cause of global disability and mortality over the past 30 years, encompassing conditions such as stroke, Alzheimer’s disease (AD), epilepsy (EP), Parkinson’s disease (PD), traumatic brain injury (TBI), multiple sclerosis (MS), spinal cord injury (SCI), and diabetic neuropathy (DN) (11, 12). Concurrently, diseases characterized by central sensitization as the core pathological mechanism, including fibromyalgia (FM), neuropathic pain (NP), and chronic fatigue syndrome (CFS), are emerging as significant challenges in both basic research and clinical translation due to their complex neuroimmune interaction mechanisms (13–16). Furthermore, numerous studies have demonstrated that the progression of neurological disorders and their associated central sensitization is often accompanied by brain function impairments, such as depression, anxiety, and cognitive deficits (10, 17–22). Neuroinflammation, a significant driver of these diseases, causes irreversible damage to the CNS, which is centered around the brain. 1his severely affects individuals’ quality of life and poses a substantial threat to society, thus emerging as a key mechanism in neuropathological diseases (23). Currently, there are no specific treatment options aimed at alleviating neuroinflammation, only symptomatic treatments are available. Moreover, due to the physiological and structural characteristics of the BBB, delivering drugs to the brain to prevent and treat neuroinflammatory lesions presents a significant challenge (24). Therefore, researching and developing non-pharmacological or non-invasive methods to establish a precise regulatory system targeting neuroimmune inflammatory damage has become an urgent need in translational medicine research.

In 2016, the National Institutes of Health (NIH) launched the “Stimulating Peripheral Activity to Relieve Conditions” (SPARC) program. This initiative aims to advance the development of novel peripheral nerve stimulation devices and modulation strategies by elucidating the anatomical and functional connectivity maps between peripheral nerves and target organs. Furthermore, it has offered new perspectives and insights for research on surface stimulation therapies, such as acupuncture (25–27). Current studies have reported that acupuncture therapy exerts significant anti-inflammatory and analgesic effects under various disease conditions by regulating neuroimmune mechanisms and related inflammatory pathways (28–31). Furthermore, it emphasizes a close biological association with the transient receptor potential vanilloid 1 (TRPV1) channel (32–36). As a pivotal nociceptor, the TRPV1 channel has had its molecular mechanisms mediating temperature and pain perception progressively elucidated since its initial identification in neurons in 1997, with related discoveries earning the Nobel Prize in Physiology or Medicine in 2021 (37, 38). Research over the past two decades has unveiled the central role of TRPV1 channels in NP and inflammatory diseases (39, 40), while confirming their critical function in transmitting acupuncture-induced cutaneous stimulation signals. Both peripheral and spinal TRPV1 channels can respond to acupuncture stimuli, mediating anti-inflammatory and analgesic effects through the activation of the autonomic nervous system (ANS) and modulation of neuroimmune interactions (33, 41). In addition to the expression of TRPV1 channels in peripheral tissues, organs, and the spinal cord, significant expression characteristics are also observed in various cell types and different regions of the brain (36, 42–45). TRPV1 channels in distinct brain areas initiate a series of immune inflammatory cascades by mediating Ca^2+^ influx in response to upstream noxious signals. This mechanism not only exacerbates the amplification of nociceptive signals but may also result in neuronal damage and dysfunction in the brain. However, compared to functional studies of TRPV1 channels in peripheral tissues and the spinal cord, there remains a gap in understanding its role within the brain. Although emerging evidence suggests that brain TRPV1 may participate in neuroinflammatory processes by regulating calcium signaling, its specific mechanisms of action and association with acupuncture modulation have yet to be elucidated.

This review will systematically explore the regulatory mechanisms of acupuncture on brain TRPV1 channels, with the aim of evaluating its immunomodulatory potential in neuroinflammatory-related diseases. The study intends to provide theoretical foundations for the development of TRPV1-targeted acupuncture therapies and to identify critical research directions that require breakthroughs in this field.

## 2 Literature retrieval and methods

### 2.1 Search strategy

This study primarily examines the mechanism by which acupuncture therapy regulates the TRPV1 channel in the brain, thereby mitigating the inflammatory response resulting from nervous system injuries. Consequently, various descriptions pertaining to “TRPV1” and “acupuncture” have been thoroughly considered. The search keyword combinations include “TRPV1/ransient Receptor Potential Vanilloid 1”, “acupuncture/acupoint/acupuncture point/meridian”, along with other relevant Chinese and English terms to ensure comprehensive retrieval of pertinent literature. Studies were sourced from PubMed, Web of Science, CNKI, and the Wanfang database, with literature selected for analysis spanning from the inception of these databases to August 30, 2025. Ultimately, a total of 22 relevant studies were chosen for analysis and review.

### 2.2 Inclusion criteria

The inclusion criteria are as follows: (1) Research Type: The study must be publicly published original experimental research, including cellular, animal, or clinical studies. Systematic reviews or meta-analyses may be referenced; (2) Subjects: Eligible subjects include cell models, neuroinflammatory animal models, or clinical patients; (3) Intervention Measures: Any form of acupuncture intervention is acceptable, including manual acupuncture(MA) and electroacupuncture(EA), with stimulation sites that may include acupoints or non-acupoints; (4) Control Measures: Control groups may consist of sham acupuncture groups, model control groups, or blank control groups; (5) Outcome Indicators: The study must measure outcome indicators related to the TRPV1 channel and neuroinflammatory markers either directly or indirectly.

### 2.3 Exclusion criteria

The exclusion criteria are as follows: (1) Non-peer-reviewed literature, such as conference abstracts, dissertations, reviews, and editorials; (2) Literature for which full text cannot be obtained or that contains incomplete data, or is unclear; (3) Literature that has been published repeatedly (if repeated, only the most complete data is included); (4) Research content that is not directly related to the ‘acupuncture-TRPV1-neuroinflammation’ pathway; (5) Studies that focus solely on the regulation of TRPV1 in neuroinflammation without involving acupuncture treatment.

### 2.4 Data extraction method

Two researchers independently extracted data using a pre-designed standardized data extraction table and conducted cross-checks to ensure accuracy. The extracted content primarily includes: (1) Basic information: first author, publication year, and journal; (2) Research characteristics: research type (*in vivo*/*in vitro*), animal or cell strain, and model establishment method; (3) Intervention details: acupuncture type, acupoint selection, and parameters (intensity, frequency, waveform, duration, and course of treatment); (4) Control settings: specific circumstances of the control group; (5) Core mechanism indicators: TRPV1 mRNA and protein expression levels, localization, use of TRPV1 antagonists or agonists, activation status of microglia and astrocytes, levels of pro-inflammatory and anti-inflammatory cytokines, and related signaling pathways (such as NF-κB and MAPK); (6) Main results and conclusions: the effect of acupuncture and the role of TRPV1 in this context.

## 3 The immunoregulatory pathway mediated by brain TRPV1 channels in neuroinflammation

**Figure 1:**
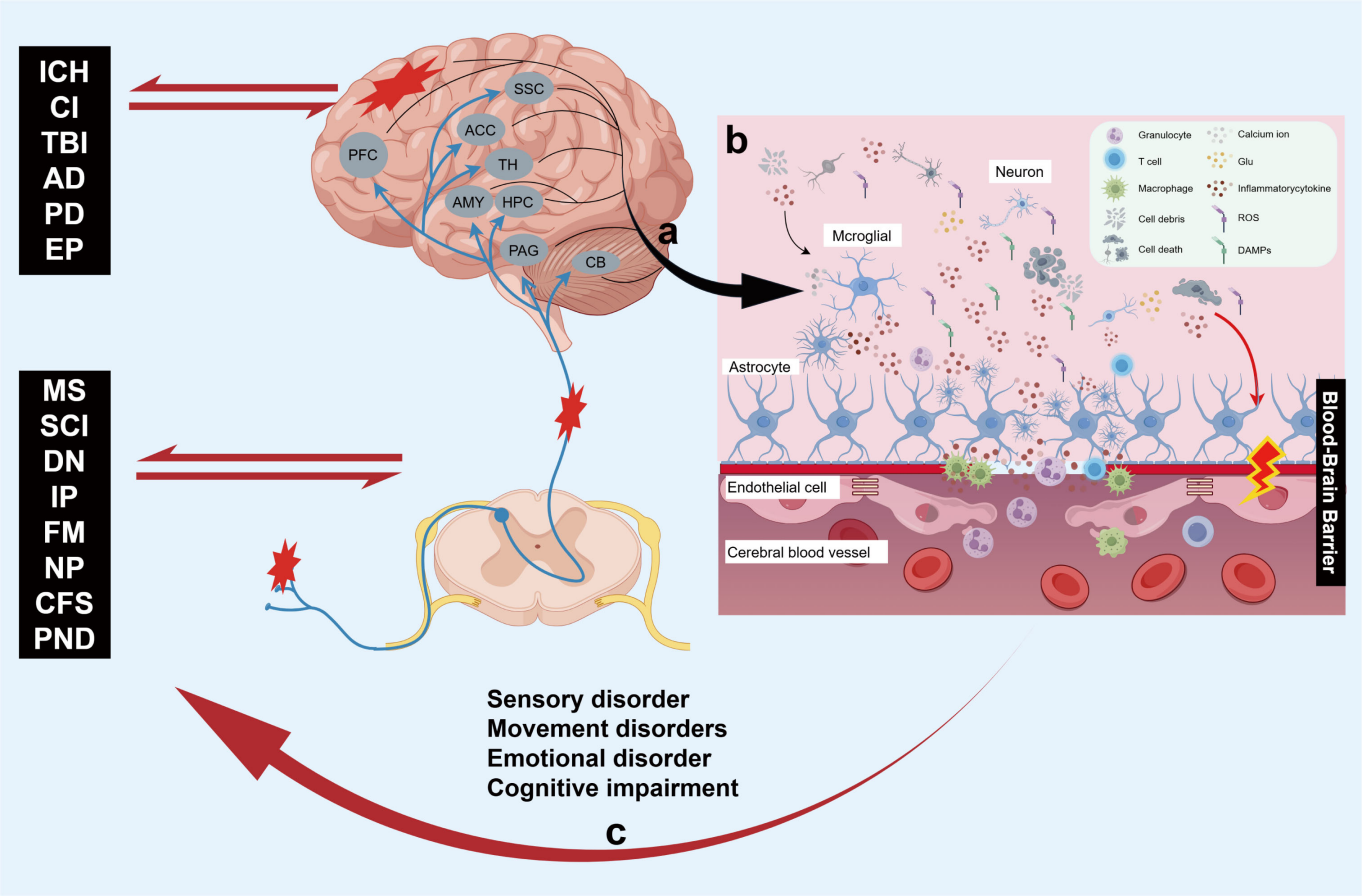
The bidirectional regulatory mechanisms between neuroinflammation and CNS lesions are complex and multifaceted. **(a)** Nociceptive signals, mediated by both peripheral and central neural pathologies, trigger neuroinflammatory responses in corresponding brain regions through a series of neural and molecular pathways. This process subsequently promotes glial cell activation across multiple brain areas, modulates the release of inflammatory mediators, and collaborates with immune cells such as T cells, neutrophils, and macrophages to drive the propagation of inflammation. **(b)** Within the pathological inflammatory microenvironment of the brain, the progressive accumulation of DAMPs, ROS, pro-inflammatory mediators, and Glu neurotoxic factors leads to cellular damage and disruption of the BBB. This disruption induces further infiltration of peripheral immune cells, exacerbating inflammatory responses and thereby forming a vicious cycle. **(c)** The persistently progressive neuroinflammatory response not only promotes the onset and progression of neurological disorders but also mediates peripheral sensitization and the generation of inflammatory pain, ultimately leading to sensory, movement, emotional and cognitive dysfunction in the brain. Created with Figdraw 2.0. (ID:WIYAY2baee).

As the center of neuro-immune regulation, the brain not only orchestrates the coordinated operation of the systemic immune response but also serves as the primary target area for the neuroinflammatory cascade. Neuroinflammation represents the central response mechanism of the CNS to pathological injury. This pathological process is characterized by the activation of glial cells, infiltration of immune cells, and a robust release of inflammatory factors, which together form a complex neuroimmune regulatory network within the brain (1). There exists a complex bidirectional regulatory relationship between neuroinflammation and neurological diseases (**Figure 1**). On one hand, nerve damage and central sensitization resulting from neurological lesions initiate an inflammatory cascade by activating central immune cells. When the nervous system is compromised, nociceptive conduction signals induced by peripheral nerve, spinal cord, and brain injuries evoke microglial phenotype reprogramming across various brain regions (**Figure 1a**). This process regulates gene transcription and the release of nociceptive mediators through oscillations in calcium signaling (46), thereby forming a complex neuroimmune interaction network with astrocytes, vascular endothelial cells, and infiltrating immune cells (such as T cells, granulocytes, and macrophages) (47, 48). This interaction activates nociceptive signaling pathways (49, 50), which drives the propagation of neuroinflammation. Conversely, the excessive production of reactive oxygen species (ROS), pro-inflammatory factors, and excitotoxic substances within the inflammatory microenvironment not only results in cellular damage and death, exacerbating the inflammatory response, but also facilitates the infiltration of peripheral immune cells and the migration of inflammatory mediators across the blood-brain barrier (BBB) by compromising its permeability. This creates a vicious cycle of ‘inflammatory damage-barrier destruction-excitation of inflammation that is challenging to interrupt (1, 51, 52), further worsening nerve damage and pathological alterations in the nervous system. Additionally, this process leads to abnormal synaptic plasticity and neuronal dysfunction in the brain (17–19, 51, 53) (**Figure 1b**). By impacting the functionality of the corresponding brain regions, it adversely affects the sensory, motor, emotional, and cognitive functions associated with these areas (1, 10, 17–21), ultimately contributing to disease progression (**Figure 1c**).

Studies have demonstrated that the activation of TRPV1 channels and the subsequent influx of Ca^2+^ play a crucial role in various aspects of neuroinflammation. Under pathological conditions, nociceptive signals from both the PNS and CNS are integrated within distinct regions of the brain through multi-level neural networks, including the peripheral nerves, dorsal root ganglia, spinal cord, and brain. This integration forms a positive feedback loop with abnormal activities generated by central sensitization, leading to an imbalance in the microenvironment of the brain. This imbalance triggers the activation of TRPV1 channels, resulting in continuous calcium influx, which participates in the activation of immune cells, the transduction of inflammatory signaling pathways, the release of damage-associated molecular patterns (DAMPs), the maintenance of redox balance, and the disruption of synaptic plasticity, among other biochemical pathways. These different mechanisms interact with one another, contributing to a persistent and exacerbated neuroinflammatory response and cell loss, ultimately causing irreversible damage to the nervous system and brain function.

### 3.1 Basic characteristics and functions of TRPV1 channel

**Figure 2:**
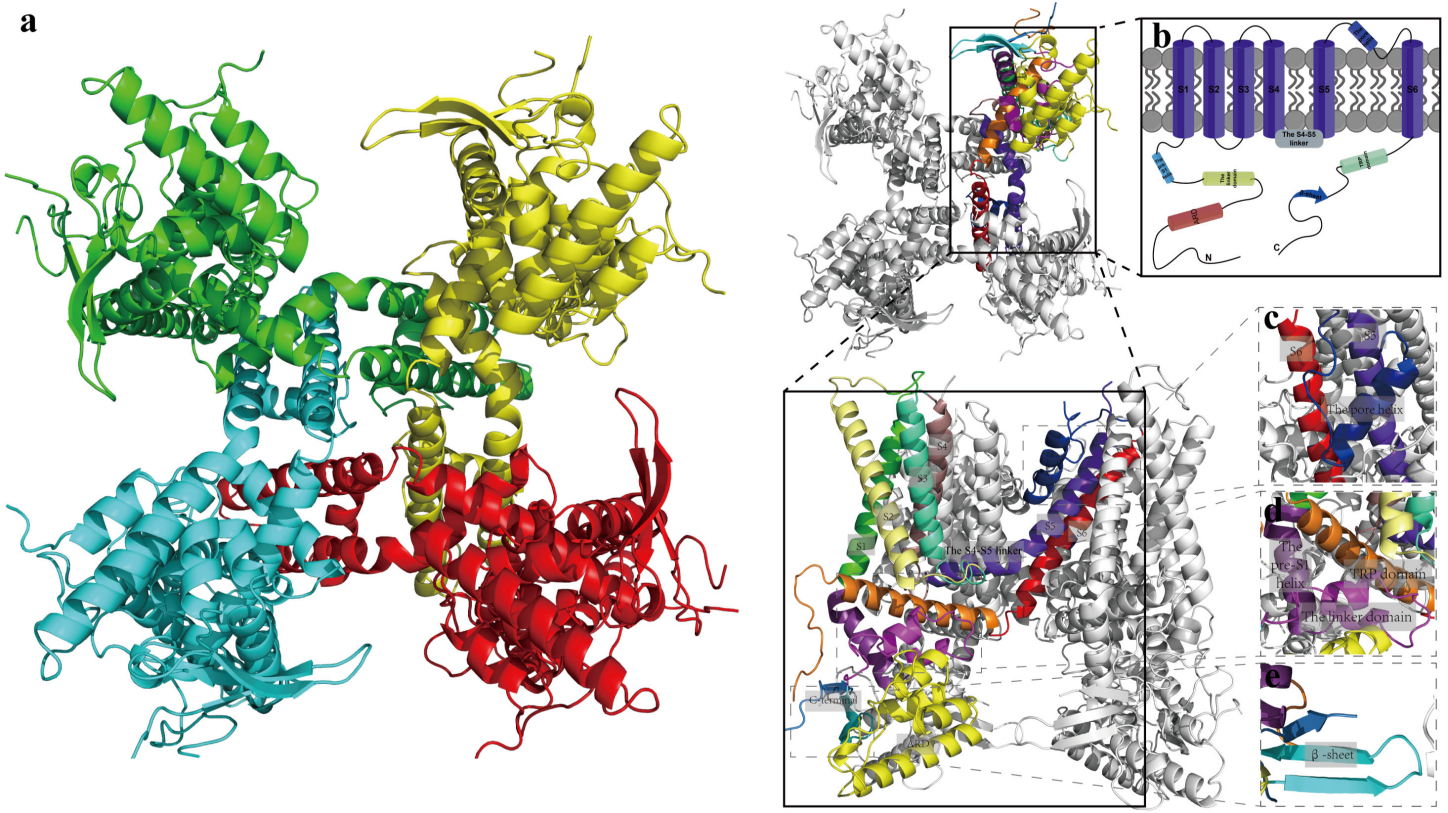
The three-dimensional structure of TRPV1 channel. **(a)** The TRPV1 channel is homotetrameric. **(b)** The pattern of functional domain of TRPV1 channel in membrane. Each monomer of TRPV1 channel composed of an N-terminal, a C-terminal and a transmembrane domain (TMD) comprising six transmembrane helices (S1-S6). **(c)** Structural details of S5, the pore helix and S6. **(d)** Structural details of the pre-S1 helix, the linker domain and TRP domain. **(e)** The β-sheet region consists of the C-terminal and a structure before ARD the N-terminal. The 3D Structure of TRPV1 channel from Pubchem database (PDB code: 7mz5); **(b)** Created with Figdraw 2.0. (ID:YAIPP5535f).

The transient receptor potential (TRP) cation channel superfamily encompasses a diverse group of ion channel proteins that are widely distributed across eukaryotic organisms. Among these, the TRPV1 channel is recognized as the first identified member of the transient receptor potential vanilloid (TRPV) subfamily in mammals. It is expressed not only in peripheral neurons, dorsal root ganglia (DRG), and the spinal cord, but also extensively in various brain regions, including the cortex, hippocampus (HPC), amygdala (AMY), thalamus (TH), midbrain periaqueductal gray (PAG), and cerebellum (CB). Furthermore, TRPV1 is found in neurons, microglia, astrocytes, and other glial cells (36, 42–45). The TRPV1 channel is a tetrameric protein composed of four identical subunits (**Figure 2a**). Each subunit consists of transmembrane domains, an intracellular N-terminus (TRPV1-N), and an intracellular C-terminus (TRPV1-C) (**Figure 2b**). These distinct structural domains possess corresponding functional characteristics, enabling the channel to play a crucial role in signaling transduction during physiological and pathological processes by responding to various chemical and physical stimuli (54–56). The transmembrane domain, an essential component of the gating mechanism, comprises six transmembrane helices. Among these, the S1-S4 transmembrane helices and the S4-S5 linker constitute conserved domains. The S5 helix, pore helix, and S6 helix collectively form a non-selective cation pore with high permeability to Ca²^+^, which regulates the entry of Ca²^+^ ions across the cell membrane in response to various stimuli (55, 57). This process mediates the inflammatory response by promoting glial cell activation and triggering a series of nociceptive pathways mediated by Ca²^+^/calmodulin-dependent protein kinase (CaMK), protein kinase A (PKA), and protein kinase C (PKC) (58–60) (**Figure 2c**). TRPV1-N consists of an ankyrin repeat domain (ARD), a linker domain, and a pre-S1 helix, which are closely associated with oxidative stress responses. Studies demonstrate that it can directly interact with Phosphatidylinositol 3-kinase (PI3K) to mediate inflammatory signaling (**Figure 2d**) (56, 61). TRPV1-C contains a TRP domain and β-sheet regions, capable of interacting with signaling proteins such as PKA and PKC, This interaction further enhances the expression of the TRPV1 channel and exacerbates Ca²^+^ influx (62, 63), and influences signal transduction pathways, including inflammatory responses, gene expression regulation, and pain perception (**Figure 2e**) (64–67).

Studies have demonstrated that the TRPV1 channel is responsive to pathological microenvironmental features, including low pH (pH < 5.9), thermal stimulation (temperatures exceeding 43 °C), infiltration of inflammatory mediators, accumulation of ROS, and stimulation by endogenous compounds such as capsaicin and cannabinoids. Through specific conformational activation, TRPV1 facilitates the transmembrane influx of cations, predominantly Ca²^+^, thereby mediating critical physiological and pathological processes. In the PNS, TRPV1 channels are predominantly expressed in the nerve terminals of primary afferent sensory neurons, which mainly consist of small- and medium-diameter neurons classified as nociceptors. When noxious stimuli act on these sensory nerve terminals, TRPV1 channels open, inducing cation influx that leads to depolarization of the nerve terminals and the generation of action potentials, thereby transmitting pain signals to the CNS, specifically the spinal cord. In the CNS, TRPV1 channels are highly concentrated in the spinal dorsal horn. Upon receiving nociceptive signals from peripheral afferents, these channels become activated, promoting Ca²^+^ influx, enhancing vesicle release, and increasing neurotransmitter secretion, which amplifies the transmission of pain signals. This process not only facilitates the formation of “central sensitization”, but also further propagates signals to higher centers of the brain. TRPV1 channels in various brain regions respond to upstream nociceptive signals, triggering a series of complex neuroimmune and biochemical cascade events mediated by Ca²^+^ influx. These events not only amplify the CNS’s processing of nociceptive signals but can also induce neuroinflammation, abnormal synaptic plasticity, and excitotoxic neuronal damage, ultimately leading to neuronal injury and impairment of brain neural functions. From the perspective of molecular mechanisms, the functional core of TRPV1 channels is centered on mediating Ca²^+^ influx. Activated TRPV1 channels primarily undergo dynamic conformational changes that create permeable pores, facilitating the entry of calcium ions and resulting in increased intracellular Ca²^+^ concentrations. This process constitutes a critical secondary messenger signal. The influx of Ca²^+^ directly initiates multi-level signal transduction pathways, including intracellular calcium oscillations, kinase phosphorylation cascades, and the activation of transcription factors. Ultimately, by regulating immune cell activity, neuro-immune interactions, the release of inflammatory mediators, and oxidative stress pathways, TRPV1 channels serve as a key molecular hub within the body’s immune regulatory network (68).

### 3.2 The immune regulation mediated by brain TRPV1 channels in neuroinflammation

**Figure 3:**
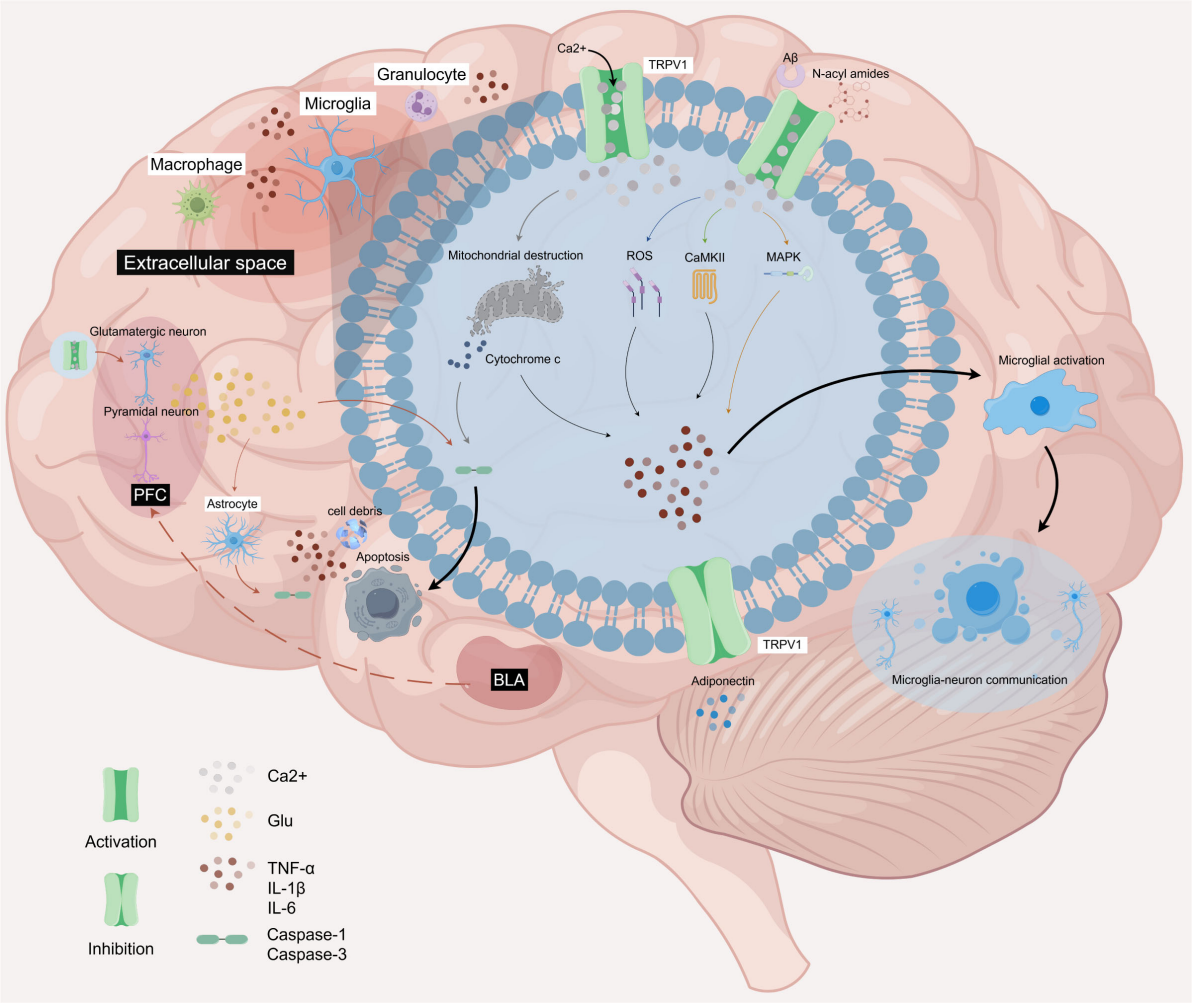
The related mechanism of microglia mediated by TRPV1 channel activation in neuroinflammation. (1) The activation of TRPV1 channel triggers Ca^2+^ influx, causing mitochondrial damage, cytochrome C and apoptosis factor release, leading to apoptosis. (2) Aβ can activate TRPV1 channel and induce ROS production in microglia. (3) TRPV1 activation can promote the activation of microglia, macrophages, neutrophils and other immune cells; (4) N-acyi amides can activate TRPV1 channel and aggravate neuroinflammation; (5) Adiponectin has anti-inflammatory and protective effects, and its inhibition can lead to the activation of TRPV1, which mediates MAPK activation and aggravates the inflammatory response. (6) BLA nociceptive signal induced activation of TRPV1 on Glu neurons and activation of pyramidal neurons in PFC cortex. By releasing Glu, glial cells released apoptotic factors, which aggravated neuroinflammation and apoptosis. (7) Activated microglia release Glu vesicles for microglia-neuron communication, aggravating the occurrence of inflammatory response. Created with Figdraw 2.0. (ID: UUTOP6279b).

The innate immune response triggered by neural injury activates immune cells, promotes the release of inflammatory factors, and initiates a series of complex cascade events that induce cellular damage and death, exacerbating the progression of inflammatory responses. Microglia and astrocytes are the primary glial cells involved in CNS inflammatory responses. Among these, microglia, which function as brain-resident macrophages, play a pivotal role in immune surveillance and the maintenance of brain homeostasis. They serve as crucial regulators of the brain’s innate immune response and are significant effector cells in neuroinflammation (69). Astrocytes, traditionally regarded as supportive cells for neurons, provide metabolic support, mediate neurovascular coupling, and regulate blood-brain barrier (BBB) permeability (47). Microglia, the primary immune cells in the CNS, play a pivotal role in driving the neuroinflammatory cascade (70), Research has reported their functional coupling with TRPV1 channels across multiple disease contexts. In 2006, Sang R. Kim’s team first demonstrated that microglia express TRPV1 channels, whose activation triggers Ca²^+^ signaling, leading to mitochondrial damage and cytochrome C release. This process activates the caspase-3 apoptotic pathway, inducing programmed cell death in microglia, and provides the first evidence that TRPV1 mediates microglial death both *in vitro* and *in vivo* (68). Subsequent studies across various disease contexts have progressively revealed the crucial role of TRPV1 channels activation in triggering Ca²^+^ signaling, which is pivotal for neuroimmune regulation centered on microglia. (**Figure 3**).

In the field of neurodegenerative diseases, the accumulation of β-amyloid (Aβ) plaques in the brain and microglia-induced neuroinflammation are considered key components of AD (71). The influx of Ca²^+^ through TRPV1 channel is recognized as a crucial ion channel for Aβ-induced microglial priming and microglial ROS production. Its pathological activation exacerbates oxidative stress and neuroinflammation in AD. Studies have demonstrated that the inhibition of TRPV1 channels can entirely eliminate Aβ-induced acute ROS production, while the inhibition of K^+^ channels does not exhibit a similar effect (72). At the level of cerebrovascular diseases, reports indicate that TRPV1 is upregulated in hemorrhagic brains and is expressed in neurons, microglia, and astrocytes. Inhibition of TRPV1 expression blocks the activation of calcium/calmodulin-dependent protein kinase II (CaMKII)-mediated downstream nociceptive pathways, ultimately reduces the activation of pro-inflammatory microglia/macrophages, decreases the number of infiltrating neutrophils around the hematoma, and attenuates the protein expression of pro-inflammatory cytokines and chemokines, as well as BBB permeability (58).

The role of central sensitization-driven brain TRPV1 channels and microglial activation in regulating systemic immune homeostasis and NP has been extensively demonstrated in numerous studies. The transmission of pain signals originates in the periphery, with the synapse between dorsal root ganglion and spinal dorsal horn neurons serving as the initial site for relaying nociceptive stimuli (73). In the context of inflammatory and NP, the activation of spinal TRPV1 channels significantly promotes glial cell activation, thereby driving the development of central sensitization (74–78). Subsequently, these signals are transmitted to the brain for further processing. Research indicates that peripheral acute inflammation induces peripheral pain and systemic inflammatory responses by driving changes in multiple N-acyl amides at the brain level, specifically within the striatum, HPC, CB, TH, midbrain, and brainstem. This process activates TRPV-mediated calcium mobilization in supraspinal microglia, triggering downstream events such as microglial activation and the release of inflammatory mediators, which ultimately lead to peripheral pain and systemic inflammatory responses (79). Furthermore, adiponectin, an adipose-derived cytokine, is not only present in serum and involved in the regulation of glucose and lipid metabolism, but studies have also shown that it can cross the BBB and enter the cerebrospinal fluid at concentrations 100-fold lower, thereby modulating the activation of immune cells in the brain and exerting anti-inflammatory neuroprotective effects (80, 81). In the partial sciatic nerve ligation (pSNL) model, research has demonstrated that adiponectin limits TRPV1 activation in the dorsal root ganglia, spinal dorsal horn, brain microglia and cortical neurons, suppresses MAPK pathway phosphorylation, and significantly ameliorates mechanical hypersensitivity associated with peripheral and central sensitization (82).

It is noteworthy that nerve injury-induced activation of TRPV1 channels exacerbates neuroinflammatory processes by modulating the interaction networks between microglia and neurons across different brain regions. The prelimbic (PL) cortex and infralimbic (IL) cortex are two crucial subregions of the medial PFC (mPFC) that play vital roles in pain processing. A study has reported that this phenomenon is associated with TRPV1 channel-triggered crosstalk between cortical neurons and glial cells: sciatic nerve injury (SNI) enhances excitatory signaling in PL-IL cortical pyramidal neurons via the basolateral amygdala (BLA)-mPFC circuit while simultaneously promoting the upregulation of TRPV1 channels in glutamatergic neurons. These combined effects lead to glutamate (Glu) overflow events in the PL-IL cortex, subsequently mediating Caspase-3 activation in microglia and the release of Caspase-1 and IL-1β factors in astrocytes, ultimately resulting in neuroinflammatory responses and pain hypersensitivity in peripheral nerve injury models (83). Meanwhile, studies have confirmed the presence of microglia-neuron communication in the ACC of the chronic constriction injury (CCI) model of the sciatic nerve, where TRPV1-activated microglia promote glutamatergic neurotransmission through the release of extracellular vesicles (EVs) (84).

In summary, the TRPV1 channel serves as a pivotal molecular mediator in the immunomodulation of the nervous system. During innate immune responses triggered by CNS injury, the activation of the TRPV1 channel initiates critical calcium signaling. This process not only directly drives the pro-inflammatory phenotypic transformation and apoptosis of microglia but also establishes a bridge for neuron-glia immune communication. In pain-related central sensitization processes, the TRPV1 channel coordinates glial cell activation and inflammatory cytokine release at both spinal and cerebral levels, thereby orchestrating the disruption of neuroimmune homeostasis and the malignant progression of neuroinflammation.

### 3.3 The inflammatory pathway mediated by brain TRPV1 channel in neuroinflammation

**Figure 4:**
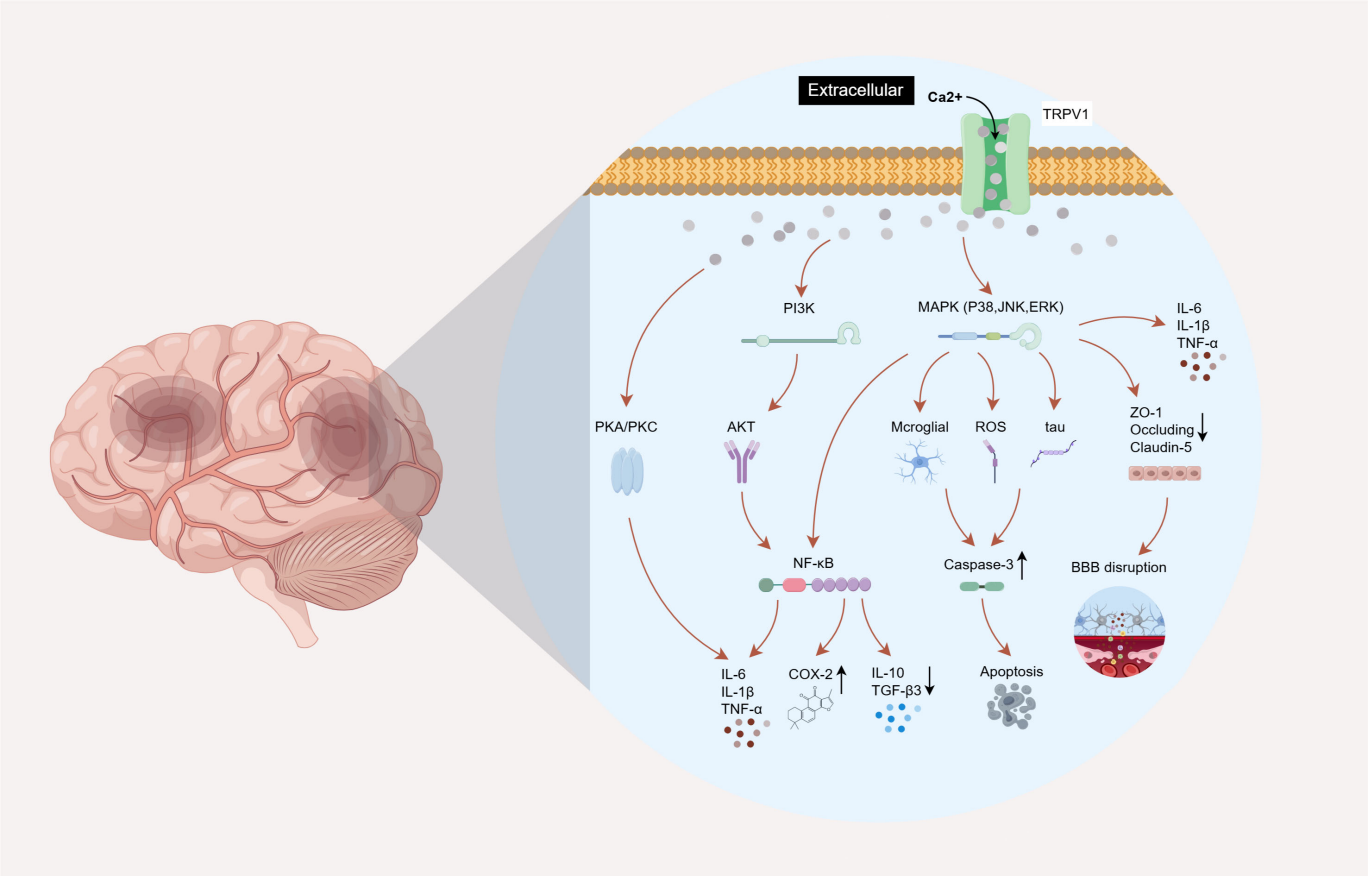
Brain TRPV1 channel activation mediated neuroinflammatory pathway. (1) TRPV1 channel-related PKA/PKC pathway phosphorylation promotes the release of inflammatory factors such as IL-1β and TNF-α, and aggravates inflammatory response and peripheral sensitization. (2) TRPV1 channel activation induces MAPK pathway phosphorylation, which is involved in microglia activation, ROS production, tau protein phosphorylation, BBB protein loss, and promotes the release of inflammatory factors such as IL-6, IL-1β and TNF-α, resulting in apoptosis and BBB destruction. (3) TRPV1 channel activation promotes the expression of PI3K/AKT pathway and MAPK pathway, activates NF-κB pathway, releases pro-inflammatory mediators such as COX-2, down-regulates the levels of protective factors such as IL-10 and TGF-β3, and aggravates the neuroinflammatory response. Created with Figdraw 2.0. (ID:RIOPY5afb1).

In neuroinflammation, the inflammatory mediators released due to immune cell activation collectively drive the neuroinflammatory cascade by triggering signal transduction within inflammatory signaling networks. Dysregulation of the TRPV1 channel-mediated Ca^2+^ influx disrupts intracellular calcium homeostasis, leading to the abnormal activation of multiple nociceptive pathways. This process involves the phosphorylation of protein kinases, such as PKA/PKC and PI3K, as well as the activation of signaling pathways, including mitogen-activated protein kinases (MAPK) and Nuclear Factor kappa B (NF-κB). These events ultimately result in cellular inflammation and cell death in the brain, causing disruption of the BBB and subsequent neuro-logical dysfunction (**Figure 4**).

The amino acid sequence of TRPV1 contains multiple phosphorylation sites for protein kinase A (PKA) and PKC. The phosphorylation of TRPV1 by PKA and PKC is closely associated with inflammatory signal transduction and the development of hyperalgesia. Furthermore, PKA and PKC can phosphorylate and sensitize the TRPV1 channel, resulting in increased Ca²^+^ influx and establishing a positive feedback loop (85–89). Studies have demonstrated that the removal of the PKC phosphorylation site (TRPV1 S801) in the TRPV1 channel of mice alleviates nociception and inflammatory pain hypersensitivity mediated by PKC phosphorylation *in vivo* (90). In the dorsal root ganglia (DRG) and spinal dorsal horn of mice with NP, elevated levels of TRPV1 have been reported to mediate the increased activity of PKC and PKA, leading to the activation of downstream inflammatory signaling pathways (91–94). Furthermore, in the context of MS, research has shown that the TRPV1/PKC pathway mediates synaptic abnormalities and neuronal loss induced by IL-1β and TNF-α in the brain (95).

MAPKs are a class of serine/threonine protein kinases that mediate inflammatory responses and apoptosis through three core MAPK subgroups: p38, c-Jun N-terminal kinase (JNK), and extracellular signal-regulated kinase (ERK) (96). Studies have demonstrated that the activation of the TRPV1 channel mediates extracellular Ca^2+^ influx in dorsal root ganglion (DRG) neurons, the spinal cord, and the brain. This process activates the MAPK phosphorylation process, triggering microglial activation, ROS production, and inducing apoptosis-mediated cascade reactions (82, 93, 97–100). In the intracerebral hemorrhage (ICH) model, decreased TRPV1 expression significantly downregulates intracellular Ca²^+^ influx and CaMKII phosphorylation, thereby blocking the P38 and JNK MAPK signaling pathways. This leads to reduced levels of factors such as IL-1β, IL-6, and caspase-3, while increasing the expression of BBB-related proteins including ZO-1, occludin, and claudin-5. Consequently, it suppresses pathological processes, including inflammatory responses, neuronal apoptosis, and BBB disruption (58, 101). The hyperphosphorylation and aggregation of brain tau protein are hallmark features of TBI, serving as risk factors for secondary brain damage, including cognitive and behavioral impairments, as well as diseases such as Alzheimer’s (102–104). Relevant studies have found that inhibiting TBI-induced TRPV1 activation can reduce tau protein hyperphosphorylation, attenuate the activation of JNK, P38, and caspase-3, and alleviate apoptosis, thereby exerting neuroprotective effects (105, 106).

The phosphatidylinositol-3 kinase (PI3K)/threonine-protein kinase AKT (protein kinase B, PKB) signaling pathway serves as a pivotal hub for cell survival signals (107). Its excessive activation can directly promote the nuclear translocation and activity of NF-κB, with both mechanisms synergistically enhancing the expression of pro-inflammatory factors and exacerbating neuroinflammatory responses. In addition to the observed activation of TRPV1 channel-regulated NF-κB and PI3K pathways in dorsal root ganglion (DRG) neurons and the spinal cord (108, 109), studies utilizing a cold stress-induced brain injury model have demonstrated that TRPV1 activation in the brain upregulates PI3K/AKT expression, promoting the production of nociceptive mediators such as IL-6, TNF-α, and NF-κB while downregulating the levels of anti-inflammatory factor IL-10 and cell growth differentiation factor TGF-β3, ultimately leading to cerebral inflammatory responses and behavioral deficits (110). The PD model further confirmed that TRPV1 inhibitors ameliorate motor dysfunction by downregulating the activity of the MAPK/NF-κB pathway in the brain striatum and reducing the expression of pro-inflammatory markers such as COX-2 (111). Notably, TRPV1 channel activation exhibits a similar mechanism in the regulation of addictive behaviors. In dorsal root ganglion (DRG) neurons, the spinal cord, and the nucleus accumbens (NAc), TRPV1 promotes the release of inflammatory factors by activating the MAPK/NF-κB pathway, mediating morphine-induced analgesic tolerance and withdrawal responses. Targeted inhibition of TRPV1 receptors can effectively block drug reward effects (112–115).

### 3.4 DAMP signaling pathway mediated by brain TRPV1 channel in neuroinflammation

**Figure 5:**
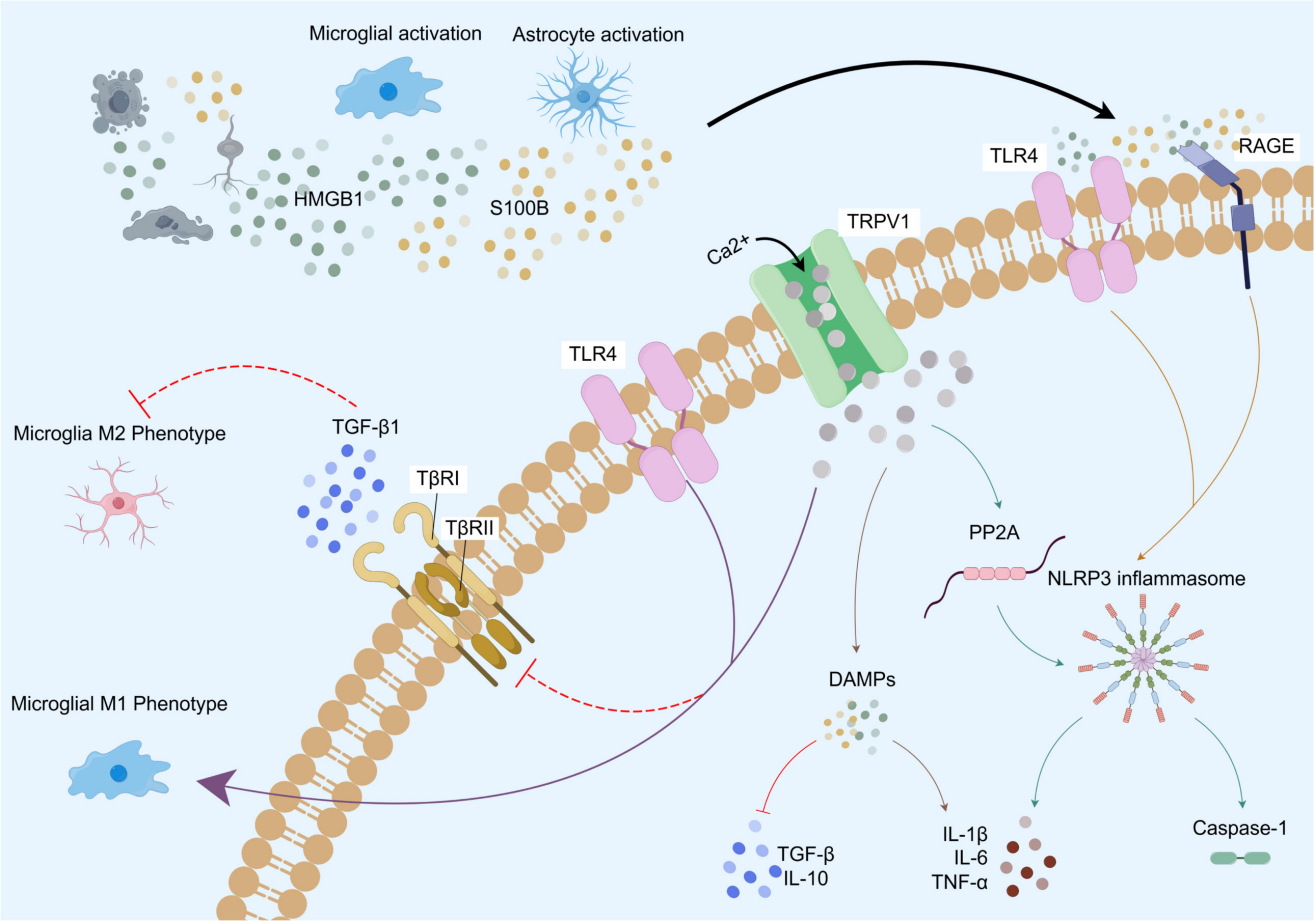
The activation of the brain TRPV1 channel mediates the DAMP signaling pathway. (1) Stress, as well as injured or necrotic cells and activated glial cells, release DAMPs such as HMGB1 and S100B. (2) The activation of the TRPV1 channel leads to Ca^2+^ influx, which promotes the release of DAMPs and up-regulates the expression of pro-inflammatory mediators. (3) Following activation, the TRPV1 channel interacts with the TLR4 receptor, inhibiting TGF-β1 signal transduction by suppressing TGF-β receptor (TβRI and TβRII) activity, thereby promoting the polarization of microglia towards the pro-inflammatory M1 phenotype while inhibiting M2 phenotype polarization. (4) The TRPV1-Ca^2+^-PP2A pathway facilitates the activation of the NLRP3 inflammasome, exacerbating the inflammatory response and increasing levels of caspase-1, IL-1β, and TNF-α. (5) Additionally, HMGB1, by binding to TLR4 and RAGE receptors, participates in the activation of the NLRP3 inflammasome, further aggravating the inflammatory response. Created with Figdraw 2.0. (ID:UYWUA7c977).

In the initiation and progression of neuroinflammation, the release of DAMPs represents a pivotal initial event that triggers and amplifies immune responses. DAMPs are a class of endogenous molecules released by stressed, injured, or necrotic cells into the extracellular space. These molecules function as “danger signals” recognized by pattern recognition receptors, such as TLR4 and RAGE, thereby activating the innate immune system (116). High Mobility Group Box 1 (HMGB1) and S100 calcium-binding protein B (S100B) are key DAMPs in the CNS. Under physiological conditions, they are predominantly localized within neurons and glial cells, but they are extensively released into the extracellular space during pathological states. Not only can they directly activate microglia, but they can also trigger potent pro-inflammatory signaling pathways through receptors such as TLR4 and RAGE, inducing the assembly of the NLRP3 inflammasome and creating a self-amplifying “inflammatory storm” (116, 117). The activation of brain TRPV1 channels is closely linked to the triggering of the DAMPs signaling cascade. TRPV1-mediated Ca²^+^ influx not only directly promotes the release of DAMPs from neurons and glial cells but also forms complex positive feedback loops with downstream DAMPs signaling pathways, collectively constituting a vicious cycle in neuroinflammation (**Figure 5**).

Current studies have indicated that the activation of TRPV1 channels, which mediates Ca^2+^ influx in dorsal root ganglion (DRG) neurons, plays a crucial role in peripheral hyperalgesia by triggering the HMGB1/S100B pathway (118, 119). Similarly, in a cold stress-induced brain injury model, the activation of TRPV1 channels promotes Ca^2+^ influx in the brain, leading to the expression and release of S100B in brain tissue. This process upregulates pro-inflammatory mediators (IL-6, TNF-α, NF-κB) and downregulates anti-inflammatory factors (IL-10, TGF-β), thereby exacerbating neuroinflammation and behavioral deficits. Notably, TRPV1 inhibitors have been shown to reverse these changes (110). In the febrile seizure model, TRPV1 activation results in elevated levels of proinflammatory cytokines, including HMGB1, IL-1β, IL-6, and TNF-α, in the HPC and cortex (120). Extracellular HMGB1 acts as a critical “alarm molecule”, predominantly activating proinflammatory responses through its receptor TLR4 (121). Further studies have revealed that the intracellular Ca²^+^ signaling activated by TRPV1 channels interacts with TLR4 to form a functional complex, collectively promoting the polarization of microglia toward the pro-inflammatory M1 phenotype while suppressing the neuroprotective M2 phenotype and TGF-β receptors (TβRI, TGF-βRII). This mechanism downregulates the expression of genes and proteins involved in transforming growth factor-β1 (TGF-β1) signal transduction and is considered a critical foundation for neuroinflammation-induced epileptogenesis (122).

The TRPV1-Ca²^+^ signaling axis serves as a critical upstream event for the activation of the NLRP3 inflammasome. The inflammasome is a cytosolic multiprotein complex that directly induces cellular damage and death by mediating the initiation and amplification of inflammatory cascades, with the NLRP3 inflammasome being the most extensively studied inflammatory complex to date (123). Current research has highlighted the pivotal role of TRPV1-NLRP3 pathway activation in the dorsal root ganglion (DRG) during peripheral inflammatory pain (124, 125). Furthermore, in the subarachnoid hemorrhage (SAH) model, it has been observed that TRPV1 channel-mediated Ca²^+^ overload in the brain activates the NLRP3 inflammasome in microglia, subsequently upregulating the expression of caspase-1, IL-1β, and TNF-α, thereby exacerbating neuroinflammatory responses in SAH (101). Further related reports indicate the potential role of the TRPV1-Ca^2+^-PP2A pathway in the activation of the NLRP3 inflammasome. Studies demonstrate that protein phosphatase PP2A is not only sensitive to changes in cytoplasmic ion concentrations (such as K+ and Ca^2+^) but also plays a significant role in the dephosphorylation process of NLRP3. The knockout of PP2A has been shown to reduce NLRP3 inflammasome activation (126, 127). In the context of MS, research confirms that microglial TRPV1 mediates Ca^2+^ influx and PP2A activity, thereby regulating NLRP3 inflammasome activation and exacerbating systemic inflammatory responses (128). Additionally, HMGB1 has been demonstrated to participate in NLRP3 inflammasome activation via TLR4 and RAGE signaling, creating a positive feedback loop that amplifies the inflammatory cascade (129).

### 3.5 Oxidative stress mediated by brain TRPV1 channels in neuroinflammation

**Figure 6:**
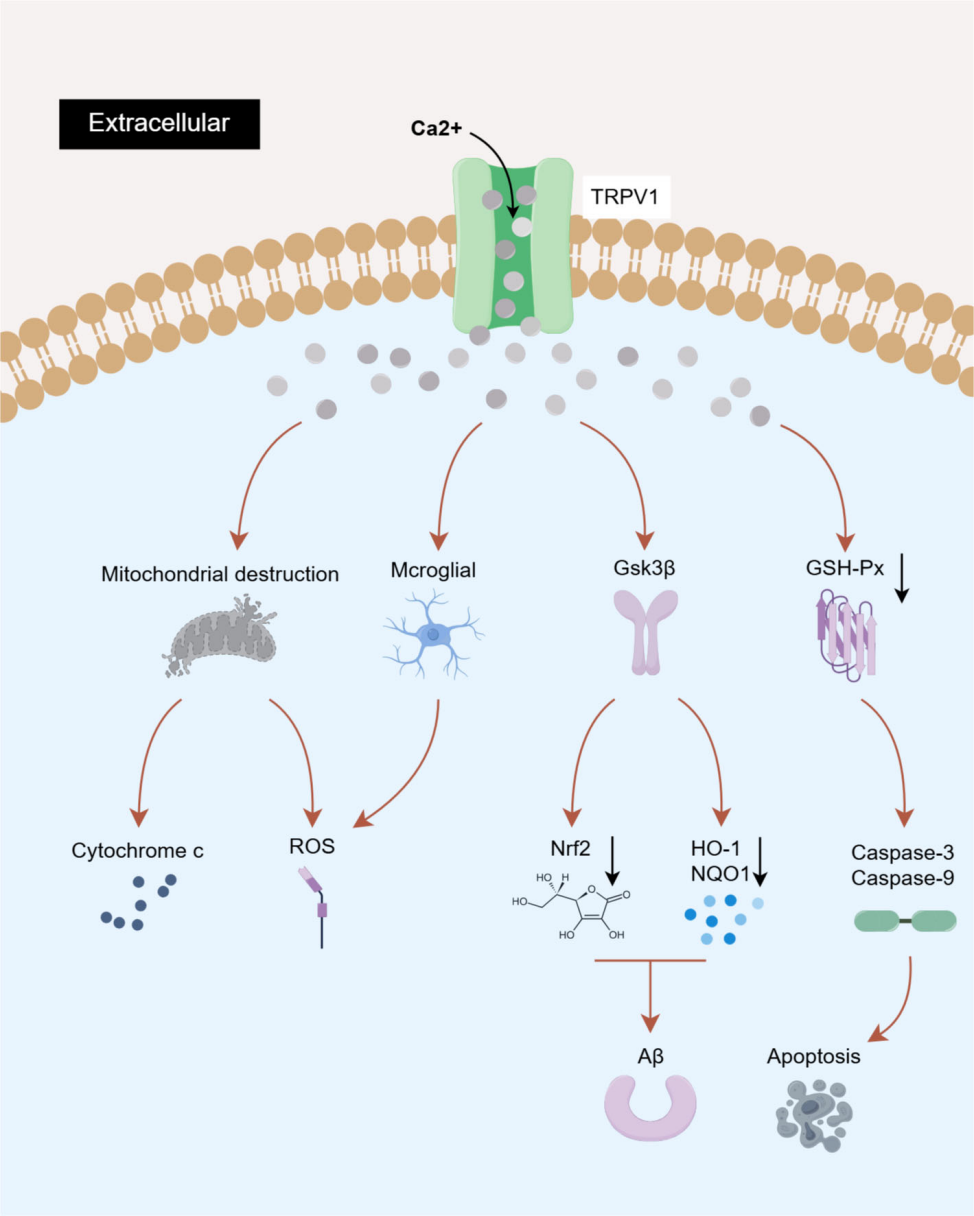
Brain TRPV1 channel activation mediates oxidative stress. (1) TRPV1 channel activation caused mitochondrial damage and cytochrome C, ROS release; (2) TRPV1 channel activation induces microglial activation and ROS production; (3) TRPV1 channel activates Gsk3β oxidative stress pathway, down-regulates the levels of Nrf2, HO-1 and NQO1 antioxidant factors, and aggravates tau protein phosphorylation; (4) TRPV1 channel activation down-regulated the expression of GSH-Px antioxidant pathway and activated the apoptosis pathway. Created with Figdraw 2.0. (ID:ITSTY45a5a).

Oxidative stress is a critical factor in neuroinflammation. Mitochondrial dysfunction and the inflammatory cascade events caused by neuroinflammation lead to a significant release of ROS in brain tissue. These processes also disrupt the redox system by suppressing the antioxidant system and activating oxidative stress pathways, collectively inducing oxidative stress. In turn, oxidative stress exacerbates cellular damage and inflammatory responses, forming a vicious cycle (130). Peripheral neuropathy and spinal cord injury-induced activation of TRPV1 channels in the dorsal root ganglia (DRG) and spinal cord results in intracellular Ca^2+^ overload. The critical role of activated oxidative stress events in central neuroinflammation and pain has been substantiated in previous studies (131–134). Relevant studies indicate that elevated levels of the oxidative stress marker lipid peroxidation (LPO) in brain tissue are closely associated with calcium overload resulting from TRPV1 activation (135). High intracellular Ca^2+^ concentrations lead to mitochondrial damage, microglial activation, and imbalance in the redox system (**Figure 6**).

The mitochondrial dysfunction and inflammatory response caused by TRPV1 channel activation, leading to excessive ROS release, constitute one of the primary causes of oxidative stress events. *In vitro* studies have demonstrated that the activation of TRPV1 leads to excessive mitochondrial Ca^2+^ loading within cells, which triggers mitochondrial damage and cytochrome C release, mediating the occurrence of cell death (68). Conversely, pharmacological inactivation of TRPV1 reduces Ca^2+^ influx and CaMKII phosphorylation, mitigates neuronal vulnerability to hemin-induced injury, inhibits apoptosis, and preserves mitochondrial integrity *in vitro* (58). This has been confirmed in cerebral hemorrhage and AD animal model, where activation of TRPV1 channels in the brain triggers increased Ca^2+^ concentration within mitochondria and mitochondrial depolarization, activates microglia, promotes massive ROS production, exacerbates oxidative stress events, aggravates apoptosis and brain damage, ultimately leading to disease progression (72, 100).

The activation of TRPV1 disrupts calcium homeostasis and, by impairing the balance of the redox system, further triggering oxidative stress events. In AD, the deposition of Aβ can promote the production of ROS and disrupt calcium homeostasis, exacerbating tau protein phosphorylation, inducing cell death, and contributing to neurodegeneration (136–139). Studies indicate that this may be associated with TRPV1-mediated Ca^2+^ influx, which activates the glycogen synthase kinase-3β (Gsk3β) oxidative stress pathway, leading to the ubiquitination and degradation of the antioxidant transcription factor E2 (Nrf2). Further studies revealed that inhibiting TRPV1-dependent calcium accumulation and the TRPV1/CaMK pathway downregulated Gsk3β activity. This, in turn, promoted the intracellular accumulation of Nrf2 and upregulated the levels of antioxidant enzymes such as HO-1 and NQO1. This mechanism reversed oxidative stress damage and amyloid-β neuropathology in AD, thereby exerting protective effects (140). Additionally, TRPV1 activation disrupts redox balance by inhibiting the antioxidant system. In cases of diabetes-induced NP, the activation of antioxidant pathways, including glutathione peroxidase (GSH-Px), has been shown to downregulate TRPV1 expression in the dorsal root ganglia and HPC. This mechanism mitigates tissue oxidative stress and apoptosis by blocking Ca^2+^ influx-induced mitochondrial damage and reducing the release of apoptotic factors such as caspase 3 and 9 (141).

### 3.6 Synaptic plasticity regulation mediated by brain TRPV1 channels in neuroinflammation

**Figure 7:**
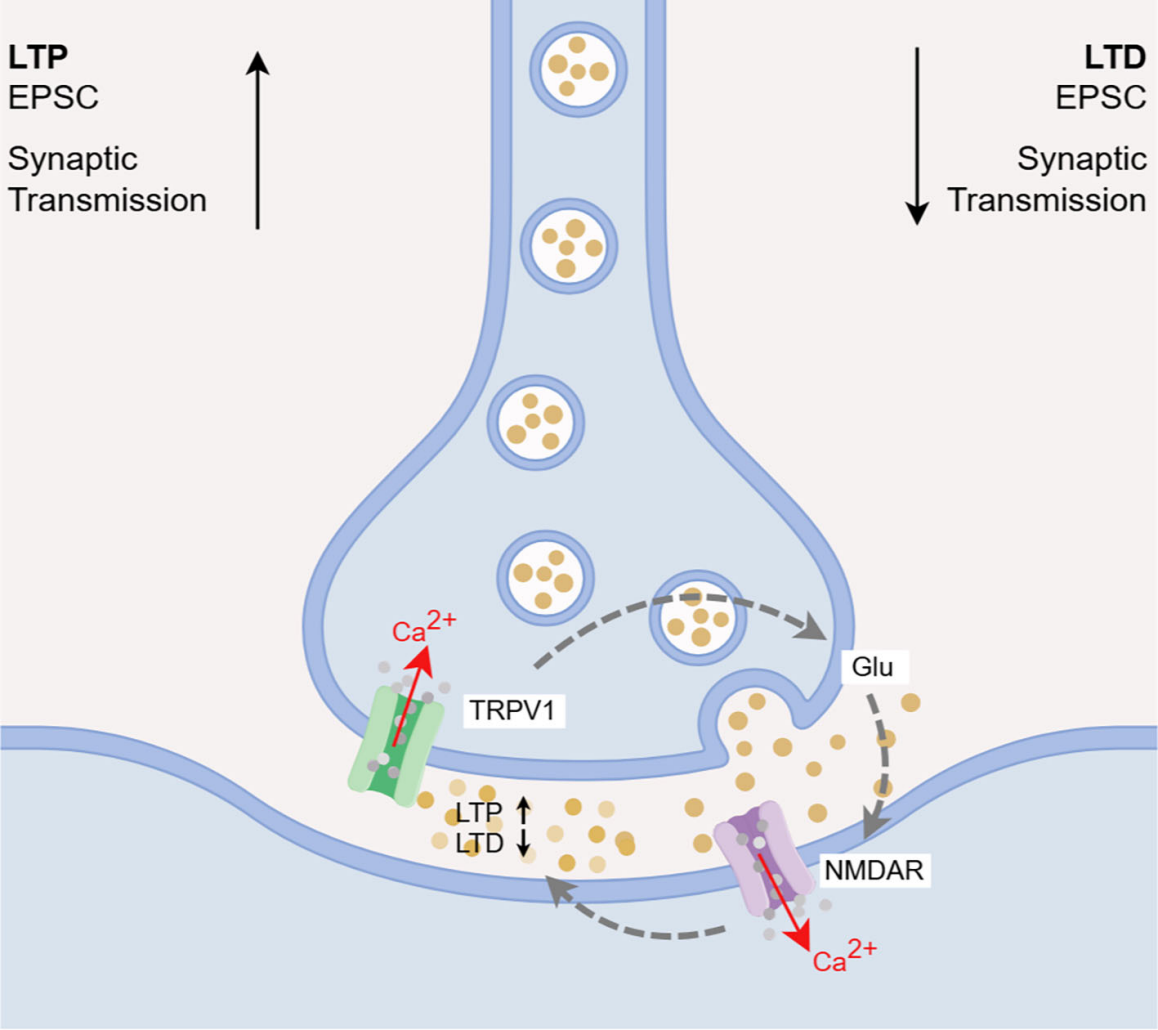
Brain TRPV1 channel activation mediates synaptic plasticity disorders. (1) TRPV1 activation promotes the release of Glu, resulting in the activation of NMDAR-based Glu receptors, resulting in neurotoxicity. (2) TRPV1 activation causes LTP/LTD imbalance, resulting in LTP enhancement and LTD inhibition. Created with Figdraw 2.0. (ID:TARRT0abd3). (3) Forming a pathway of TRPV1 activation-glutamate release-NMDAR activation-synaptic plasticity alteration.

Persistent neuroinflammation, oxidative stress, and related events lead to neuronal damage and death, resulting in dysregulation of synaptic plasticity in the brain and impairment of nervous system function. These processes are implicated in the pathogenesis of EP, PD, and Huntington’s disease, and are closely associated with functional impairments in the brain, such as mood disorders, cognitive deficits, and drug addiction (142, 143). Studies have demonstrated that TRPV1 channel-mediated calcium regulation not only modulates synaptic plasticity and nociceptive transmission at the spinal level (77, 144–146), but also participates in the pathological mechanisms of neural dysfunction across various brain regions involved in movement, cognition, and emotion. In the progression of disease, neuroexcitatory regulation mediated by brain TRPV1 channels disrupts the balance between inhibitory neurotransmitters, such as γ-aminobutyric acid (GABA), and excitatory neurotransmitters, including Glu, leading to alterations in synaptic strength characterized by long-term potentiation (LTP) and long-term depression (LTD) (**Figure 7**). This process concurrently activates glial cells, and the inflammatory factors they release further exacerbate excitotoxicity and synaptic dysfunction. Ultimately, abnormal neuronal activity, impaired synaptic plasticity, and neuroimmune responses create a vicious cycle that collectively drives the progressive impairment of brain function. Relevant studies have evaluated the effects of TRPV1 channel activation-mediated neuroinflammation on brain functions, such as mood, cognition, and drug addiction, under different contexts, confirming the association between cerebral TRPV1 channel activation and corresponding brain functional alterations across various disease conditions.

The TRPV1 channel drives synaptic excitotoxicity through the “calcium signaling-glutamate release-NMDAR activation” axis. TRPV1 is expressed in both glutamatergic and GABAergic neurons (147, 148). Glu is the predominant excitatory neurotransmitter in the CNS. Research indicates that TRPV1-mediated Ca²^+^ influx synergizes with calcium release from intracellular stores, significantly elevating local calcium concentrations and promoting microglial vesicle shedding. This process indirectly facilitates the abnormal activation of Glu receptors, primarily N-methyl-D-aspartate receptors (NMDAR), alters excitatory postsynaptic currents (EPSCs), and ultimately leads to excitotoxic neuronal damage (84, 149–151). Furthermore, it has been reported that cytokines such as IL-1β and TNF-α, secreted by immune cells, can regulate glutamate-mediated central synaptic transmission. The brain’s TRPV1 channels are implicated in glutamate-mediated central synaptic transmission and neurotoxic effects, exacerbating MS-induced synaptic dysfunction and neuronal loss (95). Additionally, research indicates that TRPV1 channels facilitate neuronal Glu release and increase EPSC frequency in brain regions such as the SSC and striatum, providing significant insights into the mechanisms underlying motor control disorders, including Parkinson’s and Huntington’s diseases (152, 153). Meanwhile, research on NP models has confirmed that pain signals can activate TRPV1 in glutamatergic neurons, resulting in extracellular Glu spillover, activation of glial cells, and ultimately leading to neuroinflammatory responses and pain hypersensitivity in models of peripheral nerve injury (83, 154).

The TRPV1-glutamate signaling directly regulates LTP/LTD, serving as the synaptic basis for learning, memory, and emotional behaviors. LTP and LTD are crucial forms of synaptic plasticity, characterized by sustained increases or persistent suppression of synaptic transmission efficiency, respectively. An imbalance between LTP and LTD leads to altered synaptic plasticity, which serves as a key mechanism underlying the hyperexcitable state observed during epileptic activity. Research indicates that TRPV1 is upregulated and activated during epileptic seizures, subsequently triggering Glu release and mediating enhanced LTP while suppressing LTD in neuronal synapses, resulting in an excitation/inhibition imbalance (148, 155–157). In TRPV1 knockout animals, weakened LTP and abolished LTD have been observed (158–160), confirming the critical role of TRPV1-mediated Glu release and alterations in synaptic strength in the process of epileptogenesis.

The functional antagonism between TRPV1 and CB1R finely regulates synaptic plasticity related to emotion and cognition. It is noteworthy that synaptic plasticity mediated by the TRPV1 channel is closely associated with anxiety and depression-like mood disorders, as well as cognitive deficits, including learning and memory impairments caused by neuroinflammatory damage. The CB1 receptor (CB1R), a core component of the endocannabinoid system, is intimately linked to GABA transmission and forms a functionally antagonistic regulatory axis with TRPV1 in the CNS (148, 161). Studies demonstrate that these two receptors exhibit significant spatial co-expression patterns in key brain regions involved in emotional regulation, such as mPFC, PAG, NAc shell, and HPC (162–167), although their neurobiological effects display functional polarization. The signaling transduction pathway activated by CB1R exerts anxiolytic and antidepressant effects by inhibiting neural activity and neurotransmitter release (168–171), whereas TRPV1 channel enhances synaptic excitability by promoting Glu release, activating NMDAR Glu receptors, and facilitating LTP. This activity increases anxiety, depression, fear-like behaviors, and impairs cognitive functions such as learning and memory (147, 160, 168–170, 172–176). The functional differentiation of these receptors based on brain region specificity provides novel molecular targets for developing precise therapeutic strategies for neuropsychiatric disorders.

## 4 Acupuncture ameliorates neuroinflammation by modulating TRPV1 channels in the brain

As a non-pharmacological intervention, acupuncture has garnered widespread attention from researchers worldwide due to its demonstrated anti-inflammatory and analgesic effects, as well as its fewer side effects and lower risk of tolerance. According to data from the NIH, over 15 million Americans have received acupuncture treatment, indicating a significant upward trend (177, 178). Currently, acupuncture therapy is extensively applied in the fields of inflammatory diseases, pain management, and mood disorders (179), with its neuroimmune regulatory effects being well-documented (180–182). Growing evidence suggests that brain TRPV1 channels serve as promising therapeutic targets for neuroimmune modulation in the CNS diseases and inflammation-related central sensitization. Recent studies have highlighted the close relationship between cutaneous stimulation signals from acupuncture and alterations in TRPV1 channels. Our research has focused on examining changes in the expression and function of TRPV1 channels at the cerebral level, as well as their mechanisms in acupuncture intervention. This work emphasizes the pivotal regulatory role of TRPV1 channels as cation channel proteins across multiple domains, including neuroprotection, pain modulation, emotional and behavioral regulation, and cognitive function. These effects involve the modulation of immune cell activation, inflammatory pathway signaling, DAMPs signaling activation, oxidative stress responses, and neurotoxicity pathways (**Figure 7**, **Table 1**).

**Table 1:** Summary of research on brain TRPV1 channel mediated acupuncture effect.

Species/model	Brain region	TRPV1 manipulation/readout	Acupuncture parameters	Main outcomes	Direction of effect	Sample size & groups	Reference
Rat, MCAO (Cerebral Ischemia-Reperfusion Injury)	HPC	TRPV1 expression downregulated; Effect reversed using capsaicin (TRPV1 agonist)	EA pretreatment, GV20, bilateral BL23, SP6, 2/100 Hz, 1 mA, total 1 hour	Reduced infarct volume, neuronal damage and apoptosis; Inhibited NF-κB signaling pathway	Protective effect (Reduced injury)	N=32; Groups: Sham, Model (MCAO), EA pretreatment (EA+MCAO), Sham EA (N-EA+MCAO)	(183)
Mouse, CFA-induced Chronic Inflammatory Pain (CIP)	DRG, Spinal Cord (SC), Thalamus, SSC	TRPV1 expression increased, decreased after acupoint injection; Validated using Trpv1 KO mice	Acupoint Injection (AI), ST36, 20% Glucose solution, 20 μl (Non-EA)	Alleviated mechanical and thermal hyperalgesia; Reduced expression of TRPV1 and related signaling molecules	Analgesic effect (Reduced pain)	N=32; Groups: Normal, CIP, AI, Trpv1-/-	(184)
Mouse, CFA-induced Inflammatory Pain	CB V, VIa, VII	TRPV1 expression increased, decreased after EA; Validated using Trpv1 KO mice	EA, ST36, 2 Hz, 1 mA, 20 min/session	Alleviated mechanical and thermal hyperalgesia; Reduced expression of TRPV1 and related molecules in CB	Analgesic effect (Reduced pain)	N=40; Groups: Control, CFA, CFA+EA, CFA+SHAM, CFA+KO (Trpv1-/-)	(185)
Mouse, SNI-induced Neuropathic Pain	DRG, SCDH, SSC, ACC	TRPV1 expression increased, decreased after EA; Validated using Trpv1 KO and chemogenetics (SSC inhibition)	EA, ST36, 2 Hz, 1 mA, 20 min/session, for 2 weeks	Alleviated mechanical and thermal hyperalgesia; Reduced inflammatory cytokines (IL-1β, IL-6, TNF-α, etc.) and TRPV1 pathway proteins; Chemogenetic inhibition of SSC produced similar analgesia	Analgesic effect (Reduced neuropathic pain)	N=45; Groups: Normal, SNI, SNI+EA, SNI+Sham EA, SNI+Trpv1-/-	(186)
Mouse, ICS-induced Fibromyalgia-like Pain	PFC, SSC, Thalamus, AMY	Trpv1 gene knockout (Trpv1^-^/^-^); TRPV1 protein measured via WB and immunofluorescence	ST36, EA, 2 Hz, 1 mA, 20 min/session, from day 3 to 5天	EA and Trpv1^-^/^-^ alleviated mechanical and thermal hyperalgesia; Reduced expression of HMGB1, S100B, TRPV1, TLR2, TLR4, RAGE and downstream molecules	Analgesic effect (Reduced pain)	N=45; Groups: Control (Con), ICS, ICS+EA, ICS+KO (Trpv1^-^/^-^)	(187)
Mouse, CSP-induced Fibromyalgia-like Pain	Hypothalamus, PAG, CB VI and VII	Trpv1 gene knockout (Trpv1^-^/^-^); TRPV1 protein measured via WB and immunofluorescence	ST36, EA, 2 Hz, 1 mA, 20 min/session, from day 5 to 7, total 3 sessions	EA and Trpv1^-^/^-^ alleviated mechanical and thermal hyperalgesia; Reduced plasma inflammatory cytokines; Decreased expression of HMGB1, S100B, TRPV1, TLR4, RAGE and downstream moleculesin hypoTH, PAG, CB; Immunofluorescence showed EA and KO reduced microglia (Iba1) and TRPV1 colocalization	Analgesic effect (Reduced pain and neuroinflammation)	N=40; groups: Normal, CSP, 2 Hz EA, Trpv1^-^/^-^	(188)
Mouse, ICS-induced Fibromyalgia-like Pain	SSC, CB V, VI, VII	Trpv1 gene knockout (Trpv1^-^/^-^); TRPV1 expression measured via WB	ST36, EA, 2 Hz, 1 mA, 20 min/session, once daily from day 4 to 8	EA and Trpv1^-^/^-^ alleviated mechanical and thermal hyperalgesia; Reduced plasma pro-inflammatory cytokines; Decreased expression of TRPV1, IL-17RA and downstream molecules in SSC and CB; Sham EA had no effect	Analgesic effect (Reduced pain and neuroinflammation)	N=40; Groups: CON, FM, EA, Sham, Trpv1^-^/^-^	(189)
Mouse, 6-OHDA-induced Parkinson’s Disease Dementia (PDD)	HPC, mPFC	TRPV1 and downstream molecules upregulated; Measured via WB/IF	EA, KI3, 2 Hz, 1 mA, 20 min/session, every other day, total 6 sessions	EA and rivastigmine reversed spatial and reversal learning deficits, decreased plasma inflammatory cytokines, downregulated TRPV1 and downstream molecules in HPC/mPFC; EA alone increased α7 nAChR and parvalbumin levels	Improved cognition, reduced neuroinflammation	N=36; Groups: Control (normal), PDD model, PDD+EA, PDD+oral rivastigmine	(121)
Mouse, ICS-induced Fibromyalgia	TH, SSC, ACC, mPFC	RPV1 and downstream molecules upregulated; Validated using Trpv1 KO mice	Acupoint Catgut Embedding, ST36, no specific freq/intensity, embedded on day 0 and 7	Acupoint embedding and Trpv1 KO alleviated mechanical and thermal hyperalgesia, reduced plasma inflammatory cytokines, downregulated TRPV1 and downstream pathway proteins in multiple brain regions	Analgesic effect (Reduced pain and neuroinflammation)	N=40; Group: Normal, FM model, FM+Acupoint Embedding, FM+Trpv1-/-	(190)
Mouse, ICS-induced Fibromyalgia	Hypothalamus, CB (Lobules V, VI, VII)	TRPV1 and downstream molecules upregulated; Validated using Trpv1 KO mice	Acupoint Catgut Embedding, ST36, no specific freq/intensity, embedded on day 0 and 7	Acupoint embedding and Trpv1 KO alleviated mechanical and thermal hyperalgesia, reduced plasma inflammatory cytokines, downregulated TRPV1 and downstream pathway proteins in hypothalamus and CB	Analgesic effect (Reduced pain and neuroinflammation)	N=40; Group: Normal, FM model, FM+Acupoint Embedding, FM+Trpv1-/-	(191)
Mouse, CFA-induced Chronic Inflammatory Pain with Comorbid Depression (CIPDC)	mPFC, Hypothalamus, PAG	TRPV1 and downstream molecules downregulated in model; Validated using Trpv1 KO mice	EA, ST36, 2 Hz, 1 mA, 20 min/session, 3 times/week	EA and Trpv1 KO alleviated mechanical and thermal hyperalgesia, improved depressive-like behavior, reduced plasma pro-inflammatory cytokines, reversed the downregulation of TRPV1 and downstream molecules in the three brain regions	Analgesic and Antidepressant effect	N=50; Group: Normal, CIPDC model, CIPDC+EA, CIPDC+Sham EA, CIPDC+Trpv1-/-	(192)
Mouse, ICS-induced Fibromyalgia-like Pain	PFC, SSC, HPC, TH	TRPV1 KO mice; TRPV1 expression upregulated (ICS-induced) and downregulated (after EA)	EA, ST36 (bilateral), 2 Hz, 2 mA, 20 min/session, consecutive 3 days (Day 3,4,5)	Alleviated mechanical and thermal hyperalgesia; Downregulated TRPV1 and related molecules in PFC, SSC, HPC, and TH	Analgesic effect (Reduced pain and neuroinflammation)	N=40; Group: Control, ICS, ICS+EA, ICS+SHAM, ICS+KO	(193)
Mouse, CFA-induced Inflammatory Pain	PFC, Hypothalamus, PAG	T TRPV1 expression increased in PFC/Hypothalamus, decreased in PAG; Reversed by EA	EA, LI4 (bilateral), 2 Hz, 1 mA, 15 min/session, consecutive 2 days (Day 2,3)	Alleviated mechanical and thermal hyperalgesia; Reversed CFA-induced protein expression changes in PFC/Hypothalamus/PAG	Analgesic effect (Reduced pain and neuroinflammation)	N=40; Groups: Normal, CFA, CFA+EA, CFA+Sham EA	(194)
Mouse, ICS-induced Fibromyalgia Pain Model	DRG, Spinal Cord, TH, SSC	TRPV1 expression increased (WB/IF); Validated using Trpv1 KO mice (Trpv1−/−)	EA, ST36 (bilateral), 2 Hz, 1 mA, 20 min/session, from day 3 to 4, total 3 sessions	Alleviated mechanical and thermal hyperalgesia; Reduced expression of TRPV1 and related pain signaling molecules; Increased PD-1 expression	Analgesic effect (Reduced pain and neuroinflammation)	N=45; Groups: Normal, FM (ICS), FM+EA, FM+PD-L1 (i.c.v.), FM+Trpv1−/−	(195)
Mouse, Acid Saline (AS) induced Chronic Pain with Comorbid Depression (CPDC) Model	CB VI, VII, VIII	TRPV1 protein decreased after AS, increased after EA; Validated using Trpv1 KO mice	EA, ST36 (bilateral), 2 Hz, 2 mA, 20 min/session, total 6 sessions	Alleviated mechanical and thermal hyperalgesia; Improved depressive-like behavior (increased open field center activity, decreased immobility in forced swim); Reversed abnormal protein expression of TRPV1 and related signaling molecules in CB	Analgesic and Antidepressant effect	N=30; Control (Con), Model (AS), EA treatment (AS+EA), Sham EA	(196)
Mouse, ICS-induced Fibromyalgia	DRG, SCDH, Hypothalamus, PAG	TRPV1 expression increased (WB/IF); Validated using Trpv1 KO mice (Trpv1−/−)	ST36, EA, 2 Hz, 1 mA, 20 min/session	EA alleviated mechanical and thermal hyperalgesia; Increased CB1 expression, decreased TRPV1 and related signaling molecules; Chemogenetic activation of PVN induced pain, inhibition of PVN alleviated pain via TRPV1 pathway	Analgesic effect (Reduced pain and neuroinflammation)	N=45; Groups: Normal, FM, FM+EA, FM+Trpv1-/–	(197)
Rat, MCAO (Cerebral Ischemia-Reperfusion Injury)	HPC	TRPV1 expression downregulated; Validated using TRPV1 antagonist AMG-517 and agonist capsaicin	EA pretreatment, GV20, bilateral BL23, SP6, 2/100 Hz, 1 mA, total 1 hour	Reduced neurological deficit scores and cerebral infarct volume; Alleviated oxidative stress and inflammation; Inhibited p38 MAPK phosphorylation; TRPV1 antagonist enhanced EA protection, agonist reversed it	Protective effect (Reduced ischemia-reperfusion injury)	N=40; Groups: Sham, Model (MCAO), EA pretreatment (EA+MCAO), Drug intervention (AMG-517/Capsaicin + EA + MCAO)	(198)
Rat, MCAO-induced Vascular Dementia	Hippocampal CA1 region	TRPV1 immunostaining showed increased expression, decreased after EA	EA, GV20, 2 Hz, 2 mA, 20 min/day, for 6 consecutive days	Improved neurobehavioral deficit scores; Restored impaired long-term potentiation (LTP); Reduced overexpression of TRPV1 and NMDAR1 (NR1) in hippocampal CA1 region	Protective effect (Improved dementia symptoms and synaptic plasticity)	N=12; Groups: Sham, Model (MCAO), EA treatment (EA)	(199)
Mouse, Acid Saline-induced Chronic Fibromyalgia (CFM)	DRG, Spinal Cord, TH, SSC	TRPV1 expression increased, decreased after EA; Validated using Trpv1 KO mice	EA, ST36, 2 Hz, 1 mA, 15 min/session, for 2 weeks	Alleviated mechanical hyperalgesia; Reduced expression of TRPV1, Nav1.7, Nav1.8 in DRG and SC; Reduced expression of TRPV1 signaling pathway molecules in TH and SSC | Analgesic effect (Reduced CFM)	Analgesic effect (Reduced pain and neuroinflammation)	N=40; Groups: Normal, CFM, CFM + 2Hz EA, CFM+sham EA, TRPV1-/-	(200)
Mouse, ICS-induced Fibromyalgia Model	DRG, Spinal Cord, Hypothalamus, PAG	Validated using Trpv1 KO mice	ST36, EA, 2 Hz, 1 mA, 20 min/session	Alleviated mechanical and thermal hyperalgesia; Enhanced CB1 expression; Inhibited microglia and astrocyte activation; Inhibited TLR4-MyD88-TRAF6 signaling pathway	Analgesic effect (Reduced pain and neuroinflammation))	N=32; Groups: Normal, FM, FM+EA, FM+Trpv1^-^/^-^	(201)
Mouse, Acid Saline-induced Fibromyalgia Model	TH, AMY, SSC	TRPV1 expression increased, decreased after EA	EA, ST35, 2 Hz, 1 mA, 15 min/session	Alleviated mechanical hyperalgesia; Reduced overexpression of TRPV1 and pERK in TH, AMY, and SSC	Analgesic effect (Reduced pain and neuroinflammation))	N=32; Groups: Control, FM model, FM+EA, FM+Sham EA	(202)
Mouse, ICS-induced FM-like Pain	TH, SSC, mPFC, HPC, CB V/VI/VII, Hypothalamus	TRPV1 KO; WB detection of TRPV1 and downstream molecules	EA, ST36, 2 Hz, 1 mA, 20 min/session (on days 15, 18, 21)	EA and TRPV1 KO alleviated mechanical and thermal hyperalgesia; Reduced overexpression of TRPV1 and related signaling molecules in multiple brain regions	Analgesic effect (Reduced pain and neuroinflammation))	N=30; Groups: CON, ICS (model), ICS+EA, ICS+Sham EA, ICS+KO (TRPV1 KO)	(203)

### 4.1 Acupuncture therapy and its protective effects in neuroinflammation

Acupuncture is a surface stimulation method that regulates bodily functions by targeting specific areas of the body known as acupoints. Since the 1970s, acupuncture has gradually gained global popularity (204). In 1990, standardized nomenclature and localization criteria for acupuncture points were established, which have since undergone continuous revision and refinement, significantly enhancing their scientific validity and authority in recent decades (205). Currently, the primary stimulation methods in acupuncture include manual needling and EA. With advancements in modern medicine, innovative techniques such as acupoint catgut embedding have been further developed. This technique involves implanting absorbable medical catgut sutures or collagen threads into specific acupoints using specialized needles, allowing for continuous stimulation of the targeted acupoints for several weeks until the sutures are completely absorbed. This method achieves prolonged anti-inflammatory and analgesic effects (31, 206). In recent decades, extensive research has demonstrated that acupuncture exerts significant neuroprotective effects in alleviating symptoms of CFS, chronic pain, ischemic stroke, PD, AD, and other neurological disorders. Furthermore, it has been shown to improve cognitive impairment, anxiety, depression, and other mental-emotional conditions by reducing inflammatory responses (207–216). Additionally, a series of studies have confirmed that the neuroinflammatory protective effects of acupuncture are associated with its modulation of multiple pathways in the brain, including immune cell activity, inflammatory signaling pathways, DAMPs signaling activation, oxidative stress, and synaptic plasticity (28, 29, 217–220).

### 4.2 Acupuncture modulates brain TRPV1 channel-mediated immune cell activity to ameliorate neuroinflammation

**Figure 8:**
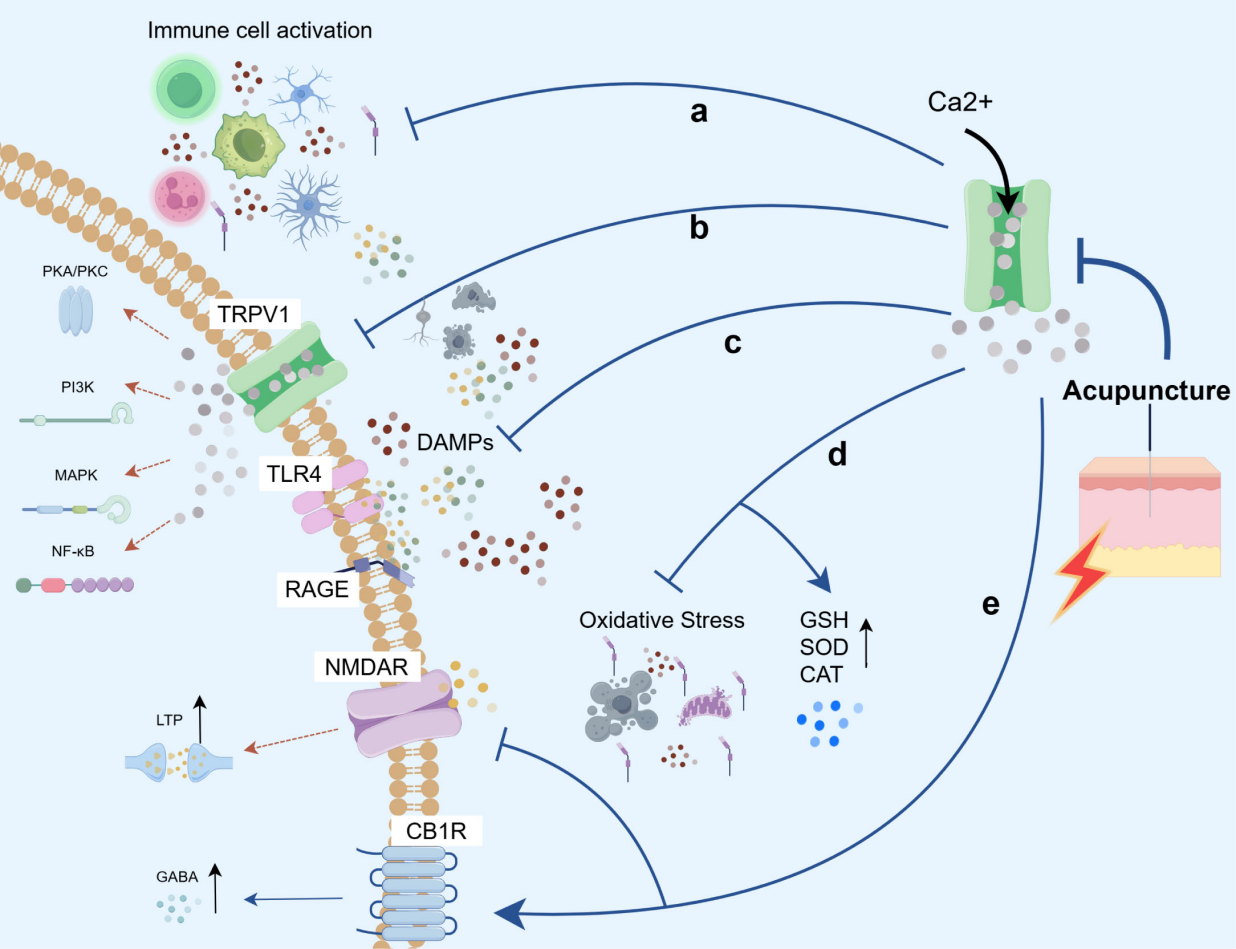
Acupuncture exerts protective effects against neuroinflammation by suppressing TRPV1 activity. **(a)**Acupuncture inhibits immune cell activation by inhibiting TRPV1 channel-mediated Ca^2+^ influx; **(b)** Acupuncture inhibits Ca^2+^ influx and the transmission of inflammatory signaling pathways by downregulating TRPV1 expression; **(c)** Acupuncture blocks the amplification of inflammatory signals by inhibiting TRPV1 channel-mediated Ca^2+^ influx, thereby attenuating DAMPs signaling and the subsequent activation of TLR3 and RAGE receptors; **(d)** Acupuncture mitigates oxidative stress and activates antioxidant pathways through TRPV1 suppression; **(e)** Acupuncture modulates the excitatory-inhibitory balance of the nervous system by regulating the transmission of excitatory and inhibitory neurotransmitters, as well as the expression of related receptors, via TRPV1 inhibition. Created with Figdraw 2.0. (ID:UTOAS41e73).

Acupuncture treatment has been shown to inhibit the upregulation of TRPV1 and glial cells in the spinal cord of mice experiencing inflammatory pain, thereby exerting anti-inflammatory and analgesic effects while reducing pain-related behaviors in these animals (221, 222). Furthermore, acupuncture is demonstrated to suppress the expression of TRPV1 channels in the brain, decrease calcium ion influx, directly regulate the activation of central glial cells—primarily microglia and astrocytes—modulate peripheral-central immune crosstalk, inhibit the release of nociceptive mediators, and exert neuroprotective effects (**Figure 8a**). Causal evidence from TRPV1 channel gene knockout and pharmacological studies strongly supports this mechanism: in the Middle cerebral artery occlusion model (MCAO), EA preconditioning significantly suppressed TRPV1 expression and glial cell proliferation in the hippocampal region, reduced neuronal apoptosis, and cerebral infarct volume. Notably, the application of the TRPV1 agonist capsaicin abolished these protective effects, confirming that acupuncture’s modulation of immune cell activity is closely associated with its inhibition of TRPV1 channel activation (183). Further studies have confirmed that acupuncture interventions in FM models downregulate the activity of TRPV1 channel receptors across several key brain regions, including the spinal cord, dorsal root ganglia, prefrontal cortex (PFC), somatosensory cortex (SSC), anterior cingulate cortex (ACC), TH, AMY, hypothalamus, PAG, and cerebellar lobules V, VI, and VII. This intervention reduces the expression of glial cell markers such as GFAP and Iba-1, inhibits downstream inflammatory cascades, alleviates neuroinflammatory responses in the brain, and improves mechanical and thermal hyperalgesia in animal models. These findings have been validated in Trpv1 gene knockout mice (184–188, 201). Furthermore, recent studies have shown that FM-induced TRPV1 activation triggers increased expression of IL-17 receptors and IL-17 factors associated with CD4+ helper T cells (Th) in the brain, further stimulating the transmission of inflammatory signaling pathways and exacerbating neuroinflammation. This pathway is blocked in Trpv1-/- mice and under EA treatment by attenuating the expression of the TRPV1-IL-17 related pathway, effectively alleviating central neuroinflammation and mechanical/thermal hyperalgesia behaviors in mice. This provides direct causal evidence for the “central TRPV1-adaptive immunity” dialogue (189).

Meanwhile, the inhibition of central TRPV1 by acupuncture can also produce systemic anti-inflammatory effects. A series of studies have analyzed relevant cellular markers and inflammatory mediators in peripheral plasma. The results indicate that acupuncture exerts anti-inflammatory effects by downregulating cerebral TRPV1 expression in neuroinflammation, thereby inhibiting the release of pro-inflammatory factors associated with peripheral immune cells, including monocytes, macrophages, and T cells. These pro-inflammatory factors include monocyte chemoattractant protein-1 (MCP-1), interferon-γ (IFN-γ), tumor necrosis factor-α (TNF-α), interleukin-1α (IL-1α), IL-1β, IL-2, IL-5, IL-6, IL-12, and IL-17, while simultaneously upregulating the anti-inflammatory factor IL-10 (121, 186, 188–192). This indicates that acupuncture coordinates systemic immune homeostasis through this central target.

### 4.3 Acupuncture modulates brain TRPV1 channel-mediated inflammatory signaling pathway to ameliorate neuroinflammation

The study demonstrates that acupuncture exerts anti-inflammatory and anti-apoptotic protective effects in neuroinflammation by modulating TRPV1 channel activation, thereby reversing abnormal intracellular inflammatory signaling pathways (**Figure 8b**). A series of studies have demonstrated that acupuncture inhibits the activation of TRPV1 channels in the dorsal root ganglion (DRG) and spinal cord (SC), maintains calcium homeostasis, and suppresses downstream kinase pathway activation to regulate pain signaling (200, 223–226). At the cerebral level, acupuncture specifically inhibits the overactivation of TRPV1 channels in neurons and microglia within key brain regions of the FM model, including the medial prefrontal cortex (mPFC), SSC, ACC, hypothalamus, HPC, TH, PAG, and CB. This action reduces Ca^2+^ influx-dependent activation of PKA, PKC, and PI3K kinases, subsequently blocking downstream MAPK and NF-κB signaling cascades. Consequently, it decreases the release of inflammatory mediators and neural damage, thereby ameliorating mechanical and thermal hyperalgesia (184–191, 193–195, 197, 200, 202, 203), as well as depressive-like behaviors (192, 196) in animal models. In the cerebral ischemia (CI) model, acupuncture exerts protective effects by downregulating TRPV1 expression in hippocampal neurons, inhibiting NF-κB signaling pathway activation, modulating the imbalance in the Bax/Bcl-2 apoptotic protein ratio, and caspase-3 release, ultimately reducing neuronal apoptosis and cerebral infarct area. Additionally, studies have shown that acupuncture suppresses TRPV1 expression in the HPC and PFC of PD mice, along with downstream inflammatory pathways such as PKA/PKC/PI3K/MAPK, alleviating neuroinflammation and improving spatial learning dysfunction and cognitive abilities in PDD mice (121).

### 4.4 Acupuncture regulates brain TRPV1 channel-mediated DAMPs signaling pathway to improve neuroinflammation

Acupuncture can effectively intervene in the overactivation of neuroimmune signaling mediated by DAMPs by inhibiting central TRPV1 channels, thereby blocking the amplification of inflammatory signals. Existing studies have reported that EA exerts anti-inflammatory and analgesic effects by suppressing the overexpression of TRPV1 in the dorsal root ganglia (DRG) and spinal cord, as well as DAMPs signaling (**Figure 8c**) (222, 224). Moreover, in FM models, acupuncture reduces the activity of TRPV1 channels in multiple central regions, including the spinal cord, dorsal root ganglia, PFC, SSC, ACC, TH, AMY, hypothalamus, PAG, and CB. This reduction decreases Ca^2+^ influx-driven immune cell activation and downstream DAMPs pathway activation, leading to lowered levels of HMGB1 and S100B (184–186). Consequently, acupuncture blocks the binding of these molecules to pattern recognition receptors such as Toll-like receptor 4 (TLR4) and the receptor for advanced glycation end products (RAGE), inhibiting key inflammatory signal amplification pathways (187, 188, 201). This mechanism ultimately ameliorates chronic mechanical and thermal hyperalgesia behaviors in pain models.

### 4.5 Acupuncture modulates oxidative stress mediated by brain TRPV1 channels to ameliorate neuroinflammation

The anti-inflammatory and analgesic effects of EA have been documented through the downregulation of inflammation-related molecules and oxidative stress markers, such as superoxide dismutase (SOD) and catalase (CAT), in the spinal cord (225). Limited evidence indicates that acupuncture offers protective effects against oxidative stress in neuroinflammation by alleviating mitochondrial damage and regulating the balance of the redox system through modulation of TRPV1 channels, which in turn reduces ROS levels (**Figure 8d**). Studies have demonstrated that EA stimulation mitigates mitochondrial damage in neural cells induced by CI through the regulation of brain TRPV1 expression and Ca^2+^ influx (183). This intervention decreases the release of cytochrome C and malondialdehyde (MDA), enhances antioxidant factors such as glutathione (GSH), SOD, and CAT, reduces levels of TNF-α and IL-1β, and ameliorates oxidative stress and inflammatory responses associated with cerebral ischemia-reperfusion injury, lowers neurological deficit scores in middle cerebral artery occlusion (MCAO) rats, and diminishes cerebral infarct volume (198, 227).

### 4.6 Acupuncture modulates synaptic plasticity mediated by brain TRPV1 channels to ameliorate neuroinflammation

Relevant studies suggest that acupuncture may enhance synaptic plasticity and reduce neuroinflammation by suppressing the expression of TRPV1 channels. This includes modulation of neurotransmitter transmission and receptor expression, which together ameliorate abnormal synaptic transmission (**Figure 8e**). A 2010 study first demonstrated that EA stimulation at Baihui (GV20) could downregulate TRPV1 overexpression induced by ischemic brain injury in the hippocampal CA1 region, alleviate glutamate-mediated neurotoxicity, and inhibit NMDAR activation, consequently improving LTP impairment and behavioral deficits (199). Subsequent studies further revealed that in a mouse model of chronic pain and depression comorbidity, acupuncture treatment targeting cerebellar regions V, VI, and VII not only downregulated NMDAR expression via the TRPV1 pathway but also enhanced the release of the inhibitory neurotransmitter GABA, alleviating mechanical and thermal hyperalgesia as well as depressive-like behaviors (196). Notably, the latest research found that acupuncture in FM mouse models, by inhibiting TRPV1 channel activation, as well as the activation of glial cells, increased CB1R expression in the dorsal root ganglia (DRG), spinal cord PAG, and hypothalamus. Consequently, this leads to a reduction in Ca^2+^ influx, suppression of neuronal excitability, and inhibition of synaptic transmission within nociceptive pathways (197, 201). These findings demonstrate that the anti-inflammatory and analgesic effects of acupuncture stimulation are closely associated with the restoration of excitatory-inhibitory balance in the CNS. However, most studies on the role of TRPV1 channels in emotional and cognitive disorders are based on animal models, with limited clinical translational evidence available to date. Dummy **Figure 8**.

## 5 Conclusions and view

Neuroinflammation is a key driver of pathological changes in the nervous system, promoting the progression of neurological disorders and causing irreversible damage to brain structure and function. This issue has garnered widespread attention from researchers worldwide. This review systematically elucidates that TRPV1 channel-mediated Ca²^+^ influx serves as a central node in the immune-inflammatory network during the pathological processes of the nervous system. At both peripheral and spinal levels, TRPV1 channels function as the “gateway” for nociceptive signal transmission and primary central sensitization. Their activation directly triggers immune responses in glial cells and facilitates the cascade release of neurotransmitters by disrupting calcium ion homeostasis, thereby amplifying inflammatory signals, promoting peripheral sensitization, and relaying these signals to the brain. At the cerebral level, TRPV1 channels induce calcium overload by responding to ascending and central intrinsic signals, which disrupts brain immune homeostasis. This subsequently activates inflammatory pathways such as PKA/PKC/MAPK, PI3K, NF-κB, as well as DAMPs pathways, triggering potent inflammatory responses and oxidative stress. These processes disrupt the central excitation/inhibition balance, collectively forming a vicious cycle that persistently exacerbates neuroinflammation and neuronal damage. This progression drives pathological changes in the nervous system and leads to functional impairments in higher brain centers governing sensation, motor control, emotion, and cognition.

Acupuncture therapy, an essential component of traditional Chinese medicine, boasts a long history and a unique theoretical system. It involves stimulating specific acupoints on the human body with specialized needles to regulate physiological functions and treat diseases, demonstrating remarkable anti-inflammatory and analgesic effects in medical practice. With the standardization of acupuncture therapy systems-including the gradual unification of acupoint localization, operational protocols, and stimulation parameters, significant progress has been made in fundamental research on its mechanisms of action (205, 228, 229). Through a comprehensive review of the pathophysiology of neuroinflammation and related disorders, as well as an exploration of the role of TRPV1 channels in neuroinflammatory regulation, we highlight the potential therapeutic efficacy of acupuncture in modulating neuroinflammation via brain TRPV1 channels across various disease contexts (**Table 2**). Firstly, it is evident that acupuncture exerts anti-inflammatory and analgesic effects by suppressing TRPV1 channel expression in peripheral nerves, dorsal root ganglia (DRG), and the spinal cord, thereby attenuating the transmission of nociceptive signals. Secondly, acupuncture alleviates the central immune-inflammatory cascade by downregulating the overexpression of TRPV1 channels in the brain. The specific mechanisms include: (1) inhibiting the activation of central immune cells and reducing the release of pro-inflammatory factors; (2) blocking Ca²^+^-dependent inflammatory signaling pathways such as PI3K/MAPK/NF-κB, thereby mitigating cell apoptosis and blood-brain barrier disruption; (3) suppressing DAMPs signaling pathways to prevent excessive activation of inflammatory responses; (4) ameliorating mitochondrial dysfunction and redox system imbalance to reduce oxidative stress levels; (5) modulating the balance of Glu and GABA neurotransmitters and the expression of NMDAR and CB1R to improve synaptic plasticity dysfunction. In summary, while acupuncture signals inhibit peripheral and spinal TRPV1 channel activity to attenuate nociceptive input, they also modulate cerebral TRPV1 channels to ‘repair the CNS’. This bidirectional synergistic regulation collectively forms the comprehensive biological basis for its systemic anti-inflammatory and analgesic effects. This not only provides insights for modulating inflammatory responses caused by nervous system injuries but also holds significant implications for the treatment and prevention of brain dysfunctions—including motor, sensory, emotional, and cognitive impairments—resulting from such injuries.

**Table 2:** The mechanism of acupuncture regulating different nervous system diseases.

Disease	Model	TRPV1-related core pathological mechanisms	Acupuncture intervention mechanism of action	Involved central nervous system regions	Related nervous system diseases & potential mechanisms of acupuncture via TRPV1	
Ischemic Stroke (173, 209–211)	MCAO	Ca²^+^ overload; Gliosis; MAPK/NF-κB pathway activation; Oxidative stress; Impaired synaptic plasticity; BBB disruption.	Inhibits TRPV1 expression, reduces Ca²^+^ influx; Inhibits gliosis; Inhibits MAPK/NF-κB pathway signaling; Alleviates oxidative stress; Restores synaptic plasticity; Reduces BBB disruption.	Cerebral cortex, HPC	Hemorrhagic Stroke (58, 100, 101)	Modulating immune cells
						MAPK signaling pathway
						Oxidative stress
						DAMPs pathway
						BBB disruption
					Alzheimer’s Disease (72, 140)	Modulating immune cells
						Oxidative stress
					TBI (105, 106)	MAPK signaling pathway
					Parkinson’s Disease (111, 152, 153, 171)	MAPK/NF-κB pathway
						Synaptic plasticity
Inflammatory Pain Hypersensitivity (and comorbid depression), Neuropathic Pain, Fibromyalgia (172, 174–178, 180–182, 204–208)	CFA, SNI, ICS	Upregulation of glial marker Iba1; PKA/PKC/PI3K/MAPKs/NF-κB pathway activation; HMGB1/S100B/RAGE/TLR4	Inhibits TRPV1 expression; Inhibits microglial activation; Inhibits inflammatory signaling pathway activation; Inhibits DAMP pathway activation;	DRG, Spinal cord dorsal horn, PFC, TH, SSC, ACC, Medial PFC, AMY, Hypothalamus, PAG, Cerebellar lobule V, VII, VII	Epilepsy (120, 147, 148, 156, 157)	Modulating immune cells
						DAMPs pathway
						Synaptic plasticity
					MS (95)	PKC pathway
						DAMPs pathway
						Synaptic plasticity
					Drug Addiction and Withdrawal (112–115)	MAPK/NF-κB signaling pathway
					Cognitive Impairment in Parkinson’s (172)	Synaptic plasticity
Parkinson’s Disease Dementia, Chronic Inflammatory Pain and Comorbid Depression (179, 182, 207)	6-OHDA, CIPDC, AS	PKA/PKC/PI3K/MAPKs/NF-κb; Impaired synaptic plasticity	Inhibits TRPV1 expression; Inhibits inflammatory signaling pathway activation; Improves synaptic plasticity; Exerts anti-inflammatory effects, improves cognitive function and depressive behavior	HPC, mPFC, Hypothalamus, PAG	Memory Deficits in Alzheimer’s (174)	MAPK/NF-κB信号通路
					Anxiety, Depression, Fear-like Emotional Disorders (160, 168–170, 172, 175, 176)	Synaptic plasticity

**Figure 9:**
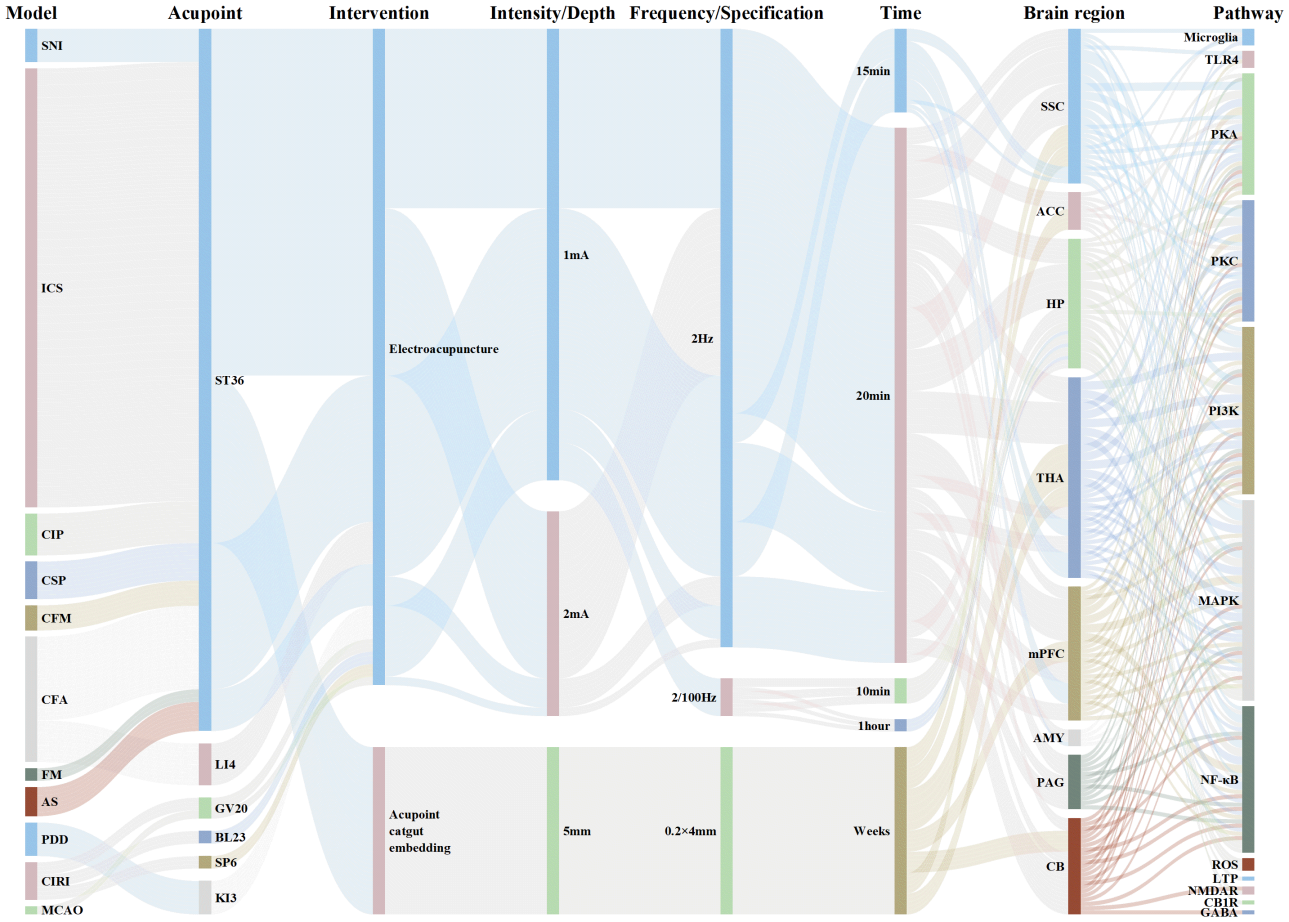
Stimulation parameters and related mechanisms of acupuncture intervention in different disease models. To explore the therapeutic mechanism and parameter rules of acupuncture regulating TRPV1 channel, including acupoint selection, acupuncture intervention method, intensity/frequency of EA, depth/specification of acupoint catgut embedding, to evaluate its immunomodulatory potential in neuroinflammation-related diseases model such as SIN (175), Intermittent cold-stress (ICS) (176, 178, 180, 181, 204, 206, 208, 223, 229), CIP (172), Cold stress pain (CSP) (177), CFM (191), Complete freund’s adjuvant (CFA) (174, 182, 205), AS (207, 228), PDD (179), Cerebral ischemia reperfusion injury (CIRI) (173, 209), MCAO (211), and to provide a basis for the development of targeted acupuncture therapy. By Origin 2024.

This study systematically reviews the potential mechanisms through which acupuncture modulates neuroinflammation via the TRPV1 channel in the brain, highlighting its therapeutic potential. However, several important limitations and unresolved questions persist in this field, which will provide critical directions for future research:

1. The ascending regulatory mechanism remains unclear: it is still not fully understood how the physical stimulation of acupuncture on the body surface is converted into biological signals and transmitted to the brain through specific neural or humoral pathways, precisely regulating the expression and function of TRPV1. Studies have demonstrated that EA can downregulate TRPV1 channel expression and reduce the release of inflammatory factors while simultaneously increasing the levels of α7 nicotinic receptors and parvalbumin (121). This suggests its potential activation of the α7 nicotinic acetylcholine receptor (α7 nAChR)-mediated cholinergic anti-inflammatory pathway (CAIP) (230). However, the relationship between brain TRPV1 channels and α7nAChR in the anti-inflammatory effects of acupuncture necessitates further investigation.
2. Acupoint specificity and complex dose-effect relationships: Although acupuncture therapy is widely applied, controversies persist regarding its acupoint specificity and the dose-effect relationships of stimulation parameters. **Figure 9** summarizes the currently reported acupoints, stimulation parameters, and corresponding mechanisms in studies investigating TRPV1 channel modulation in the brain via acupuncture. Currently, research in this field predominantly focuses on pain-related studies, while investigations into neurodegeneration remain at a preliminary stage. The selection of acupoints is primarily concentrated on specific points such as ST36, but whether they possess specificity still requires verification through acupoint-sham point controlled trials. Furthermore, the quantitative relationship between acupuncture parameters (combinations, frequency, intensity) and TRPV1 channel gating characteristics (such as desensitization/sensitization thresholds and calcium-dependent inactivation) remains unestablished. Additionally, there is a lack of precise regulatory strategies based on neuroanatomical and functional connectivity. In the future, it will be necessary to establish a standardized mapping system for “stimulation parameters-biological effects” by utilizing electrophysiology and calcium imaging techniques to facilitate precise interventions.
3. Biphasic and context-dependent functions of TRPV1 channels: It is noteworthy that while this review primarily focuses on the pro-inflammatory role of TRPV1 channels in neuroinflammation, studies have demonstrated that TRPV1 expression is reduced in specific brain regions in certain disease models, where it instead exerts protective effects. These protective effects are associated with its role in mediating autophagy regulation (231–241). Research indicates that mild to moderate autophagy can maintain CNS homeostasis, whereas sustained or excessive autophagy may lead to increased accumulation of autophagosomes in the cytoplasm, degradation of essential components, or even trigger autophagic cell death or other forms of cell death mechanisms (242). Acupuncture therapy is believed to alleviate disease symptoms through the bidirectional regulation of autophagy, although its underlying mechanisms remain insufficiently explored (243). This suggests that the role of TRPV1 channels in the nervous system is not singular; rather, their ultimate effects may form a functional continuum dependent on key factors such as disease stage, cell type, and microenvironment. Future research should focus on identifying the molecular switches (e.g., specific phosphorylation sites or interacting proteins) that transition TRPV1 channel function from “pro-inflammatory” to “protective”, thereby providing a foundation for developing highly selective and context-adaptive regulatory strategies.
4. Challenges in clinical translation and precision medicine: Currently, most evidence is derived from animal models, with extremely limited human data available. Future research urgently requires validation through clinical imaging techniques, such as fMRI and PET, or through biomarker detection studies. Ultimately, studies should integrate multiomics technologies, neural circuit tracing, and clinical data to analyze individual differences based on the TRPV1 signaling network. This integration aims to advance acupuncture from ‘empirical medicine’ to a new level of ‘precision medicine’ that is tailored to patients’ genetic backgrounds, disease types, and neuroimmune statuses.

## Glossary

CNS: Central nervous system
PNS: Peripheral nervous system
AD: Alzheimer’s disease
EP: Epilepsy
PD: Parkinson’s disease
TBI: Traumatic brain injury
ICH: intracerebral hemorrhage
CI: cerebral ischemia
MS: Multiple sclerosis
SCI: Spinal cord injury
SAH: Subarachnoid hemorrhage
PND: peripheral nerve disorders
DN: Diabetic neuropathy
FM: Fibromyalgia
NP: Neuropathic pain
CFS: Chronic fatigue syndrome
IP: inflammatory pain
NIH: National Institutes of Health
SPARC: Stimulating Peripheral Activity to Relieve Conditions
TRP: transient receptor potential
TRPV1: Transient receptor potential vanilloid 1
ARD: ankyrin repeat domain
ANS: autonomic nervous system
ACC: Anterior cingulate cortex
SSC: Somatosensory cortex
PFC: Prefrontal cortex
mPFC: medial prefrontal cortex
PAG: Periaqueductal gray
HPC: Hippocampus
TH: Thalamus
AMY: Amygdala
CB: Cerebellum
ROS: Reactive oxygen species
BLA: the basolateral amygdala
Glu: Glutamate
Aβ: β-amyloid
TLR4: Toll-like receptor 4
TGF-β1: Transforming growth factor-β1
TβRI: TGF-β receptor I
TβRII: TGF-β receptor II
pSNL: Partial sciatic nerve ligation
SIN: Sciatic nerve injury
ICS: intermittent cold-stress
CIP: chronic inflammatory pain
CSP: cold stress pain
CFM: chronic fibromyalgia
CFA: complete freund’s adjuvant
AS: Acid-Saline
CCI: Chronic constriction injury
EVs: Extracellular vesicles
MAPK: Mitogen-activated protein kinases
NF-κB: Nuclear Factor kappa B
PKA: Protein kinase A
PKB: protein kinase B
PKC: Protein kinase C
JNK: c-Jun N-terminal kinase
ERK: extracellular signal-regulated kinase
CaMKII: Calmodulin-dependent protein kinase II
PI3K: Phosphatidylinositol 3-kinase
NAc: Nucleus accumbens
LPO: Lipid peroxidation
Gsk3β: glycogen synthase kinase-β
Nrf2: Antioxidant transcription factor E2
GSH-Px: Glutathione peroxidase
GABA: γ-aminobutyric acid
LTP: Long-term potentiation
LTD: long-term depression
NMDAR: N-methyl-D-aspartate receptor
EPSCs: Excitatory postsynaptic currents
CB1R: CB1 receptor
EA: Electroacupuncture
MA: Manual acupuncture
CIRI: cerebral ischemia reperfusion injury
MCAO: Middle cerebral artery occlusion
DMAPs: Damage-associated molecular patterns
MDA: Malondialdehyde
SOD: superoxide dismutase
CAT: catalase
CAIP: cholinergic anti-inflammatory pathway
ST36: Zusanli
LI4: Hegu
GV20: Baihui
BL23: Shenshu
SP6: Sanyinjiao
KI3: Taixi
α7 nicotinic acetylcholine receptor: α7 nAChR
